# Sources of Black Carbon Deposition to the Himalayan Glaciers in Current and Future Climates

**DOI:** 10.1029/2018jd029049

**Published:** 2018-06-27

**Authors:** Matthew J. Alvarado, Ekbordin Winijkul, Rebecca Adams-Selin, Eric Hunt, Christopher Brodowski, Chantelle R. Lonsdale, Drew T. Shindell, Gregory Faluvegi, Gary Kleiman, Thomas M. Mosier, Rajesh Kumar

**Affiliations:** 1 Atmospheric and Environmental Research, Lexington, MA, USA; 2 Nicholas School of the Environment, Duke University, Durham, NC, USA; 3 Center for Climate Systems Research (CCSR), Columbia University, New York, NY, USA; 4 NASA Goddard Institute for Space Studies, New York, NY, USA; 5 World Bank Group, Washington, DC, USA; 6 Advanced Study Program, National Center for Atmospheric Research, Boulder, Colorado, USA; 7 Atmospheric Chemistry Division, National Center for Atmospheric Research, Boulder, Colorado, USA,

## Abstract

WRF-Chem and a modified version of the ECLIPSE 5a emission inventory were used to investigate the sources impacting black carbon (BC) deposition to the Himalaya, Karakoram, and Hindu Kush (HKHK) region. This work extends previous studies by simulating deposition to the HKHK region not only under current conditions, but also in the 2040–2050 period under two realistic emission scenarios and in three different phases of the El Niño-Southern Oscillation (ENSO). Under current conditions, sources from outside our South Asian modelling domain have a similar impact on total BC deposition to the HKHK region (35–87%, varying with month) as South Asian anthropogenic sources (13–62%). Industry (primarily brick kilns) and residential solid fuel burning combined account for 45–66% of the in-domain anthropogenic BC deposition to the HKHK region. Under a no further control emission scenario for 2040–2050, the relative contributions to BC deposition in the HKHK region are more skewed toward in-domain anthropogenic sources (45–65%) relative to sources outside the domain (26–52%). The in-domain anthropogenic BC deposition has significant contributions from industry (32–42%), solid fuel burning (17–28%), and diesel fuel burning (17–27%). Under a scenario in which emissions in South Asia are mitigated, the relative cotribution from South Asian anthropogenic sources is significantly reduced to 11–34%. The changes due to phase of ENSO do not seem to follow consistent patterns with ENSO. Future work will use the high-resolution deposition maps developed here to determine the impact of different sources of BC on glacier melt and water availability in the region.

## 1 Introduction

The Himalaya, Karakoram, and Hindu Kush (HKHK) mountain ranges run 2,400 kilometers through six nations (India, Pakistan, Afghanistan, China, Bhutan, and Nepal) and contain 60,000 square kilometers of ice, which is more ice than any other region outside the Poles. The combination of snowmelt, icemelt, and rain from these mountains forms the source of the Indus, Ganges, and Brahmaputra, and six other large rivers in Asia. These mountain ranges are also a region in flux, having already experienced an increase in annual mean surface temperature of 1.5 °C relative to the pre-industrial average (Stocker, 2013). As a result, Himalayan glaciers have been retreating during most of the past century (Gautam et al., 2013).

More than 750 million people live within the Indus, Ganges, and Brahmaputra basins, including an estimated 200 million living in the headwater regions (Shresta et al., 2015). People residing in rural areas largely derive their livelihoods from agriculture, which is a vital source of food for those residing in the urban communities of the region. Water derived from the headwaters also contributes significantly to energy production (e.g. Mirza et al., 2008; Gautam et al., 2013). While the origin of water availability (i.e. snowmelt, glacier melt, and rain) differs within and between each of the study basins, snowfall and glacier melt are important controls on the timing and availability of surface water at many locations within the basins, especially the headwater regions (Immerzeel et al., 2010; Lutz et al., 2014). The combined changes to water availability and climate change have the potential to have multifaceted economic impacts, including on the aforementioned agriculture and energy production sectors, as well as on human health (Gautam et al., 2013).

Melting glaciers, loss of seasonal snow and precipitation changes could pose significant risks to the stability of water resources in the South Asia region. Glaciers help to moderate river flows in the region’s major rivers by providing a source of meltwater in hot, dry years and storing water during colder, wetter years. The dependence on glaciers and snow make these rivers particularly vulnerable to climate change. Well before a global annual average warming of 2 °C is reached, a rapid increase in the frequency of low snow years is projected, with a consequent shift towards higher winter (DJF) and spring (MAM) runoff (World Bank, 2013).

While there is evidence that South Asia’s water towers are threatened by climate change, the impacts on glaciers, water availability, and food security may differ substantially among basins and cannot be generalized (Immerzeel et al., 2010). It could be that the effects of climate changes on the Indus and Brahmaputra basins will be more severe because a larger percentage of these basins are glaciated and more of their water falls in the mountain portion of the basin, creating greater dependency on seasonal melt. On the other hand, recent climate changes have not impacted snowfall in the Indus Basin as greatly as in the surrounding basins due to the atmospheric circulation patterns in the region (Kapnick et al., 2014).

In addition to threats from global climate change, black carbon produced and circulated within the region is both increasing the absorption of solar radiation by the glaciers through decreasing the glacier surface’s reflectance and raising air temperatures, which also increases melt. In these ways, black carbon is becoming a significant factor in the retreat of some Himalayan glaciers (Bond et al., 2013; Ramanathan & Carmichael, 2008; World Bank, 2013). The timing and quantity of precipitation has the potential to affect the mass balance of glaciers and snow cover, and may have a larger influence on hydrology than glacial melt alone (NRC, 2012). Absorbing aerosols – including black carbon – in northern India during late spring (May-June) might advance rainy periods (Meehl et al., 2008) and decrease late monsoon (July-August) precipitation (Lau & Kim, 2006, 2010; Meehl et al., 2008).

In this project, the Weather Research and Forecasting model coupled with Chemistry (WRF-Chem; Grell et al., 2005), a coupled meteorological and chemical transport model (Section 2.1) driven with detailed emission inventories (Section 2.2), was used to understand the impacts of regional emissions and transport on the wet and dry deposition of black carbon to the HKHK region under current conditions and under future climate projections for 2040–2050. WRF-Chem has been used multiple times to successfully simulate trace gas and aerosol emissions and distribution over the HKHK region (e.g., Kumar et al., 2012a, 2012b, 2013, 2014, 2015a, 2015b; Ghude et al., 2013; Jena et al., 2014). Specifically, Kumar et al. (2012a) used radiosonde, infrared sounder (AIRS), and Tropical Rainfall Measuring Mission (TRMM) observations of temperature, water vapor, tropopause pressure, and precipitation to evaluate a meteorological-only WRF simulation over South Asia during 2008 and found it was “of sufficient quality for use in chemistry simulations.”

Tagged black carbon (BC) tracers (e.g., Kumar et al., 2015a) from several BC emission source sectors and countries were used to assess their relative impact on BC deposition in a single model run. In this approach, BC from different source sectors is “tagged” by having the model simulate a set of species that have the physical and chemical properties of BC, but are emitted, transported, and deposited separately so the impacts of the different sectors on the final BC deposition can be calculated. Kumar et al. (2015a) used tagged tracers of BC within WRF-Chem to identify the dominant sources of BC in South Asia during the 2006 Integrated Campaign for Aerosols, Gases, and Radiation Budget (ICARB), but did not analyze the relative impacts on BC deposition. The data from this study will be used in a companion study to estimate the impact of BC emissions on the current and future albedos of the glaciers in the HKHK region, and the resulting impacts on water resources in South Asia.

## 2 Methods

In this study, 28-day (14-day spin-up, 14-day analysis) WRF-Chem simulations were run for each season (January – Winter Monsoon; April – Monsoon Transition; July – Summer Monsoon; and October – Monsoon Transition) for both current conditions (represented using the moderate ENSO year of 2013) and three future years between 2040 and 2050 – a moderate ENSO year, a La Niña year, and an El Niño year. Each of the future cases was run using two emission scenarios, giving a total of 7 simulated years (see Table 1). The choice of a 14-day spin-up differs from the 20-day spin-up used by Kumar et al. (2015a). Our choice was made after determining that a 14-day spin-up was sufficient to reduce the tagged BC from initial conditions to less than 1% of the total BC at the end of the spin-up period. We also chose to simulate the year of 2013 rather than 2006 (as in Kumar et al., 2015a) as 2013 was more recent (and thus more relevant to current policy) and was also a neutral year for the ENSO index, whereas 2006 started in a moderate La Nina and ended with moderate El Nino, and thus could mix the impacts of the ENSO cycle with the seasonal cycle.

### 2.1 Modeling Configuration

**Figure 1.**
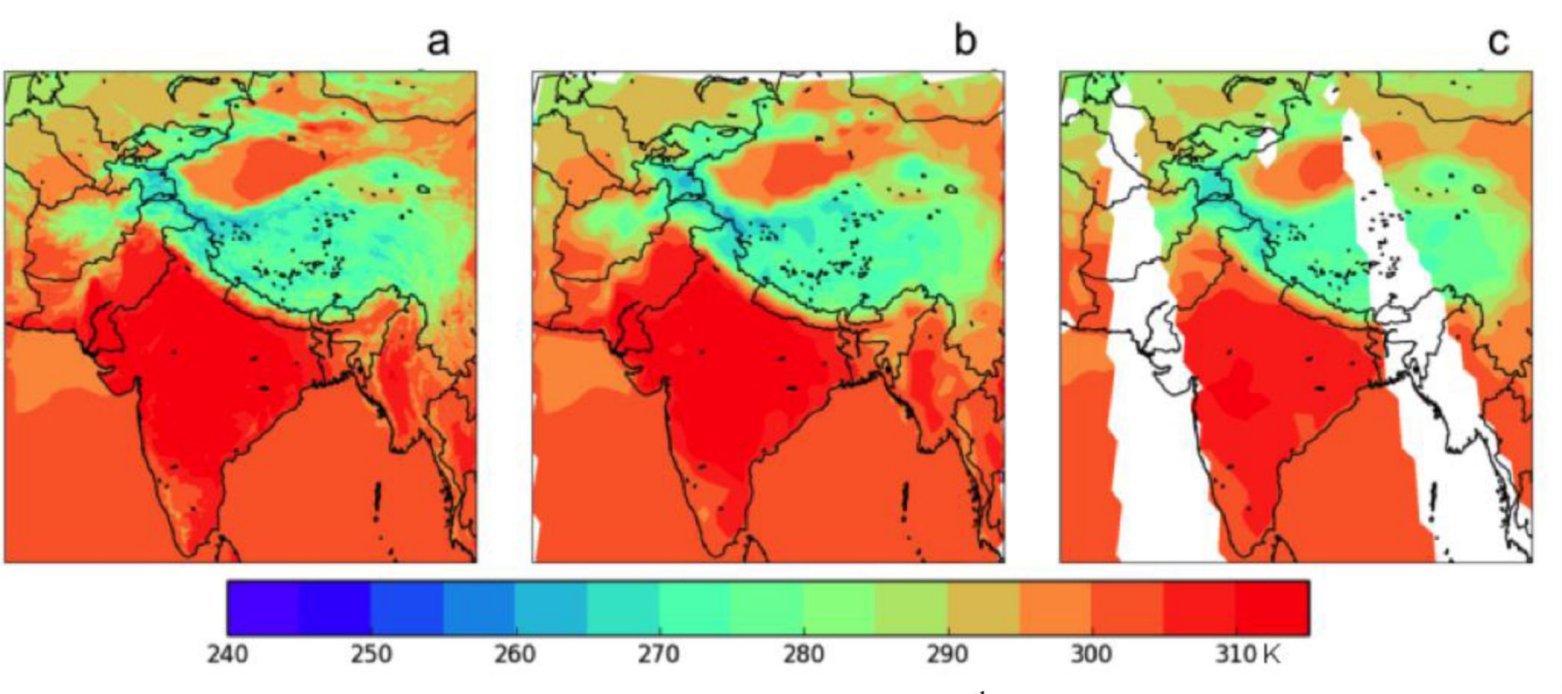
(a) WRF 2m Surface Temperature (K) on April 27^th^ at 08:30 Z (13:30 LST) on the native 12 km grid. (b) As in (a), but interpolated to the 1^o^ × 1^o^ grid of the AIRS data. (c) AIRS surface air temperature retrieved on the daytime (ascending) orbit on April 27^th^ at ~13:30 LST.

WRF-Chem is an internationally recognized model collaboratively developed among the scientific community (Grell et al., 2005; Fast et al., 2006). The chemistry portion of the model is fully coupled with the meteorological portion, meaning the chemical and meteorological processes are allowed to feedback to each other. WRF-Chem v3.6.1 was run with the GOCART bulk aerosol scheme over a 12 km horizontal resolution grid that covers India and the HKHK region (Figure 1a) and with 35 vertical layers. This resolution was chosen to help provide finely resolved BC deposition information to a future study that will use a hydrology model with a resolution of approximately 1 km. While a 12 km resolution may still be too coarse to fully resolve the complex topography of the HKHK region, this was the finest resolution that was possible given the computational resources available for this work. The selected WRF-Chem parameterizations are listed in Table 2, and generally follow those used in Kumar et al. (2015a). The United States Geological Survey (USGS) terrain and land-cover classification datasets were used at a 30-second resolution, with the WRF Preprocessing System (WPS) interpolating the dataset to this domain. The GOCART configuration of WRF-Chem was used because it minimized the computational cost while preserving the ability of the model to simulate aerosol-radiation interactions. Combustion sources are assumed to emit hydrophobic BC aerosols that then become hydrophilic with an e-folding time of 1.2 days as in Chin et al. (2002). The grid-scale wet deposition parameterization in WRF-Chem was used, which only simulates in-cloud scavenging of the BC, not the impaction or below-cloud scavenging, and thus may underestimate the wet deposition. These assumptions are evaluated by examining the model’s ability to simulate surface BC concentrations in Section 3.1 below. Our aerosol model is thus similar to that used in the GEOS-Chem study of Kopacz et al. (2011) on the impact of BC deposition on the glacier albedo of the HKHK region, except that, as noted above, our configuration does not account for impaction or below-cloud scavenging.

For the current condition (2013) runs, meteorological initial boundary conditions were derived from the NCEP FNL (Final) Operational Global Analysis data (NCEP, 2000) while chemical initial and boundary conditions were taken from the NCAR MOZART-4/GEOS-5 global simulations (Emmons et al., 2010).

For the future year scenarios, both sets of initial and boundary conditions were taken from full transient simulations from 2010–2100 of the GISS-E2-R model (Schmidt et al., 2014) for RCP 4.5 and RCP 4.5 except South Asian emissions mitigation cases using the methods of Trail et al. (2013). We chose to simulate RCP 4.5 as this pathway assumes that much of the world is taking moderate actions to reduce their BC emissions, and thus represents a future state where the influence of South Asian sources should be relatively more important. We felt that this RCP scenario thus resulted in more policy-relevant results for the nations of South Asia.

The GISS-E2-R model runs were performed using physics option 3, which includes prognostic aerosols and aerosol-cloud interactions with cloud microphysics, which allows the GISS-E2-R model to fit into the “Group 1” models identified by Wang (2015) as best simulating the impacts of anthropogenic aerosols on climate. From these simulations, three years in the 2040–2050 range were selected for downscaling: one El Niño year, one La Niña year, and one climatologically average year (based on temperature excursions from the decadal mean averaged over the Nino3.4 region; 5S-5N; 120–170W). For each year, two simulations were performed (Table 1) - one that followed RCP 4.5 precisely, and one that followed RCP 4.5 except that South Asian aerosol emissions were removed to be more consistent with the emission mitigation scenario (Section 2.2).

Tagged tracers for BC were added to the WRF-Chem code following the methods used by Kumar et al. (2015a). These tagged tracers track BC aerosol emissions from 5 sectors (Diesel Fuel, Industry, Solid Fuel, Open Burning, and Biomass Burning) and 6 nations (India, China, Nepal, Pakistan, Bangladesh, and Myanmar), as well as BC from the initial and boundary conditions. The emission sources included in these categories are discussed in Section 2.2.

### 2.2 Emission Inventories

The emission inventories used in this research were the Representative Concentration Pathways (RCPs; RCP, 2016), the ECLIPSE inventory of Stohl et al. (2015), and the residential combustion inventory of Winijkul et al. (2016) and Winijkul & Bond (2016). RCP 4.5 (significant with feasible actions) provides emissions of BC and co-emitted pollutants (i.e., SO_2_, NO_x_, NH_3_, VOC, OC, CO, CH_4_) from 2010 to 2100, and our future emission projections were scaled to be consistent with this pathway (see below).

The ECLIPSE emission dataset (Stohl et al., 2015) and the Winijkul et al. (2016) dataset (hereafter called the “HOUSEHOLD” dataset) were used to disaggregate total emissions to different emission sectors and geographic regions (at the state level within India and China, and at the country level for other countries).

- The ECLIPSE dataset provides emissions of short-lived climate pollutants (SLCPs) based on current legislation (i.e., current and planned environmental laws, considering known delays and failures up to now but assuming full enforcement in the future; see Stohl et al., 2015) from the year 2010 to 2050. The current version (ECLIPSE 5a) has been substantially updated to include flaring emissions from oil and gas production, high emitter engines, and wick lamps, in addition to previously available sources such as Euro II standard diesel engines, brick kilns, and open burning. The inclusion of gas-flaring emissions improves the match with observed BC concentrations (European Commission, 2016). The ECLIPSE inventory is available at 0.5^o^ × 0.5^o^ horizontal resolution with geographical emission location information at the state level for India and China and at the country level for other Asian countries.
- The HOUSEHOLD dataset provides emissions from residential combustion in 2010 by utilizing GIS data to allocate population and resource availability (i.e., fuelwood, electricity). This inventory uses the night light product from NASA satellites to identify access to electricity and other datasets to identify access to fuelwood. Recent emission measurements of different stove technologies, including BC from wick lamps, are also included. The spatial distribution of residential combustion emissions in this dataset takes different approach from that in ECLIPSE as the emissions are calculated and allocated based on the urban/rural classification, spatial distribution of fuel use, and the fuel and stove technology combination. This inventory is available at a 0.05^o^ × 0.05^o^ horizontal resolution for all countries in Asia.

In this research, the ratio of the RCP 4.5 pathway to that of the ECLIPSE 5a current legislation (CLE) scenario was used to scale the combined ECLIPSE and HOUSEHOLD inventories for all scenarios, so that the RCP 4.5 pathways provided the rate of change of the total emissions while the ECLIPSE and HOUSEHOLD datasets provided data on emissions from each source type and the spatial distribution of emissions. The ratios of the RCP 4.5 to ECLIPSE 5a CLE projections were calculated separately for each source category (e.g., transportation, energy, industry) and for each pollutant.

The emission source groups in this study were developed based on the emission activities in the ECLIPSE inventory. In this study, the industry source group includes emissions from industrial process, industrial combustion, and brick production. Brick kilns are an important component of this combined industrial source, accounting for 89.5% or more of the total (annual average) industrial emissions of BC in the ECLIPSE database for 2010 for India, Nepal, and Bangladesh.

The diesel source group includes emissions from on-road diesel vehicles, i.e., cars, buses, light-duty vehicles, and heavy-duty vehicles, but not diesel generators and other uses of diesel fuel. Emissions from agricultural waste burning were included in this study as the open burning source group. For the solid fuel source group, household emission from cooking, heating, and residential boiler activities using fuelwood, agricultural waste, other biomass (such as dung), and coal as fuel sources were included.

Two scenarios for future anthropogenic emissions (Stohl et al., 2015) that cover the range of minimum and maximum emission reductions were examined in this work:

- The no further control (NFC) scenario uses the same assumptions as current legislation until 2015 but assumes no further legislation is introduced subsequently, even if currently committed. This leads to higher emissions than in CLE for most pollutants, and after our scaling approach results in higher emissions than the RCP 4.5 scenario.
- The ECLIPSE mitigation (MIT) scenario that includes all measures with beneficial air quality and climate impact. These measures range from replacing kerosene wick lamps with LED lamps to providing cleaning cooking stoves for the residential sector. The emission reduction calculations for all pollutants included in this MIT scenario are consistent with the approach presented in UNEP/WMO (2011) and Shindell et al. (2012).

These realistic scenarios were chosen, rather than fixed perturbations (i.e., ±50%), as this makes the results of this study more directly relevant to policy makers and the general public.

Wildfire emissions of BC and other pollutants were estimated using v1.5 of the Fire INventory from NCAR (FINN, Wiedinmyer et al., 2011) and distributed vertically using the online plume-rise module (Freitas et al., 2007). The current conditions (2013) and future moderate ENSO runs both used the same biomass burning emissions from 2013, as that was a recent moderate ENSO year. For the future La Niña case, biomass burning emissions from the 2011 La Niña were used, while for the Future El Niño case biomass burning emissions from the 2015 El Niño were used. While this approach ignores the potential changes in biomass burning emissions as climate changes, as well as the interannual variability in biomass burning emissions, it provides a consistent natural BC background for assessing the changes brought about by emission changes in the different anthropogenic sectors. Dust emissions were simulated using the Air Force Weather Agency (AFWA) scheme in WRF-Chem (Adams-Selin, 2012).

We also note that there is an inherent uncertainty in these emission estimates, as the inventories all depend on estimates of fuel use, emission factors, and other parameters that are themselves highly uncertain. While we have not performed a full uncertainty propagation of the BC emission inventories used here, we assume that these estimates are uncertain to a factor of 2 in the absolute emissions (about twice the range of the different BC inventories studied by Crippa et al., 2016), which would imply an uncertainty of about a factor of 2 in our estimates of BC deposition. The uncertainty in the fractional contributions of different source types and regions is harder to quantify. For example, we would expect the errors in biomass burning emissions to be uncorrelated with errors in the anthropogenic emissions, but we would expect errors in the emissions estimates for different regions of South Asia to be highly correlated, as they were derived from consistent datasets as discussed above. In the absence of more exact estimates, we thus suggest that our estimates of the fractional contribution of different sources of BC to the BC deposition in the HKHK region are uncertain to about a factor of two as well.

## 3 Validation of 2013 Runs Against Observations

As the WRF-Chem model has been extensively evaluated against available observations of BC, atmospheric temperature, and precipitation in previous studies (e.g., Kumar et al., 2015a, 2015b), this section briefly compares our results to datasets in those previous studies to identify any major differences between our simulations and this previous work. Data from the full 28-day simulations are used below to better compare with monthly-average or longer datasets on BC concentrations and precipitation, but no significant differences between the spin-up and analysis periods were noted for these variables.

### 3.1 BC surface concentrations

The geographic and seasonal distribution of BC aerosols and deposition in 2013 as simulated in this study is consistent with previous studies of BC in South Asia (e.g., Kopacz et al., 2011; Kumar et al., 2015a, 2015b). For example, Table 3 assesses the ability of the above modelling configuration to successfully simulate BC surface concentrations in South Asia by comparing our mean April results (28 days) with the March-May surface observations and WRF-Chem results of Kumar et al. (2015a). Our model simulations generally capture the geographic variation of BC over South Asia. The largest discrepancies are the model underestimates at Dibrugarh, Port-Blair, and Lhasa. These sites are also underestimated in the study of Kumar et al. (2015a), but the underestimate is larger for our simulation. However, this study does a better job than Kumar et al. (2015a) of matching observed concentrations at Dehli, Langtang, and NCO-P. As the WRF-Chem configuration used here was similar to that of Kumar et al. (2015a), as were the sources of chemical and meteorological boundary conditions, the differences between the simulations are likely primarily due to differences in our anthropogenic emissions inventories, and secondarily to the fact that Kumar et al. (2015a) simulated 18 March to 11 May 2006, while we simulated 1 April to 28 April 2013.

The seasonal cycle of the simulated black carbon concentrations is also consistent with the simulations of Kumar et al. (2015b) and the observations shown therein. Table 4 summarizes the seasonal results for the stations listed in Table 3. As expected, the modeled BC concentrations peak in January during the winter monsoon (dry season) and are at a minimum in July during the summer monsoon (wet season), except at Trivandrum, which instead has a minimum during the monsoon transition periods. This cycle is generally followed in the observations for the lower elevation sites, but the two HKHK sites with seasonal data (Nainital and NCO-P) have a maximum observed concentration in April, instead of in January. Thus we conclude that the model has reasonable skill in predicting current surface BC concentrations in South Asia.

### 3.2 Surface Temperature

The ability of our modelling configuration to accurately simulate the surface temperature over South Asia was assessed by comparing our modelling results for the surface (2 m) air temperature with the daily Level 3 gridded (1^o^ latitude by 1^o^ longitude) daytime (~13:30 local solar time) and night-time (~01:30 local solar time) surface temperature observations of the Atmospheric Infrared Sounder (AIRS) satellite instrument (AIRX3STD, V006, downloaded from the NASA Giovanni website on March 27, 2017). As our WRF output was available for 00Z, 06Z, 12Z, and 18Z, corresponding to 05:00, 11:00, 17:00, and 23:00 local time, the output results were linearly interpolated in time to match the AIRS overpasses. Example plots for April 2013 are shown in Figure 1. The model generally does a good job of capturing the variation of surface temperature over South Asia, including the rapid transition from the warm interior of India to the much cooler temperatures in the Himalayas and Tibetan plateau. However, the model overestimates the surface temperature within India for several days of the simulation, including that in Figure 1. We conclude that our modelling configuration is able to represent the variation of surface temperature over South Asia with reasonable accuracy.

### 3.3 Surface Precipitation

**Figure 2.**
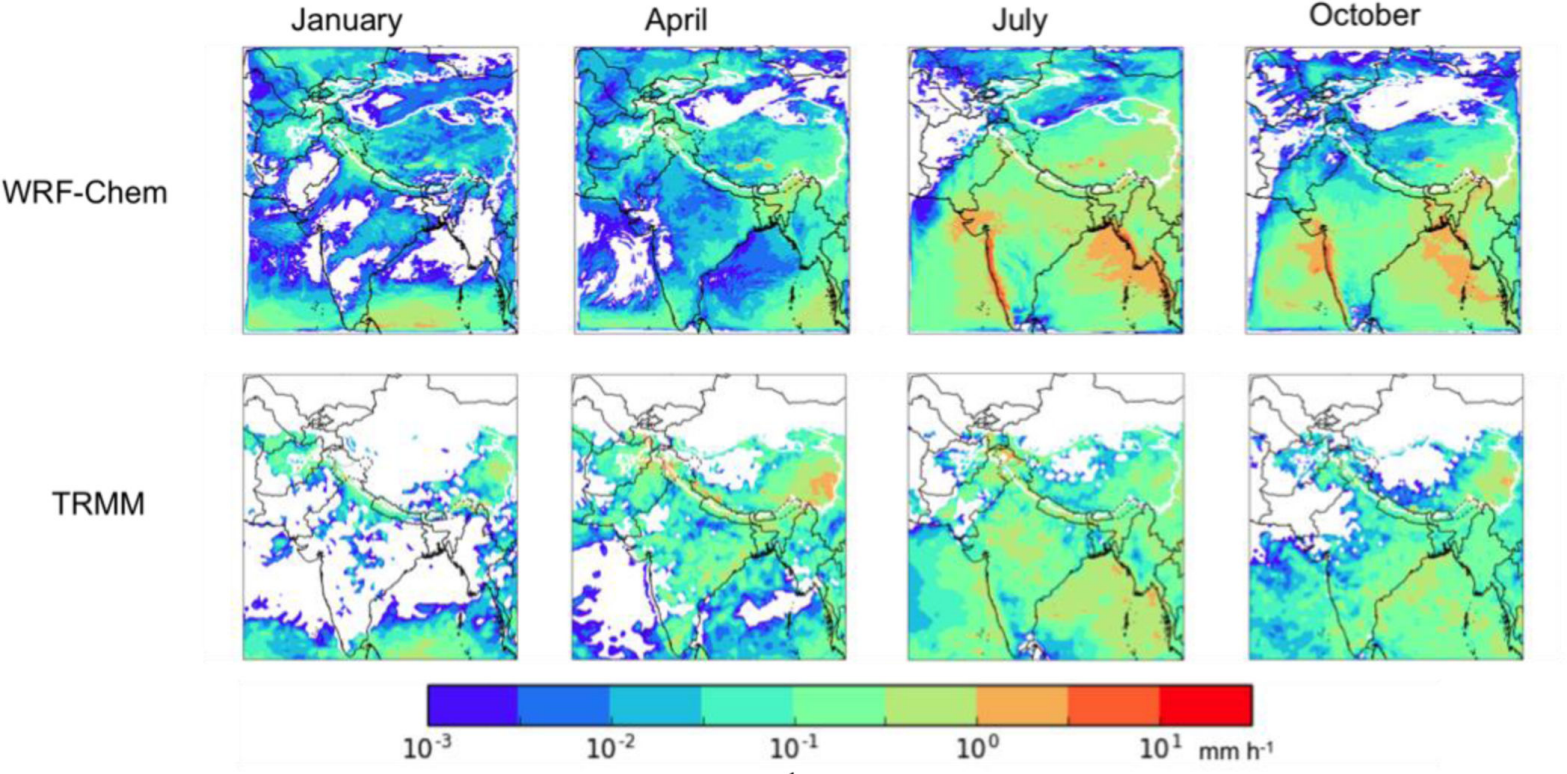
(top) Mean WRF precipitation (mm h^−1^) for (left to right) January, April, July, and October 2013. (bottom) TRMM monthly mean precipitation data for the same months.

The ability of our modelling configuration to simulate the accumulated surface precipitation was assessed by comparing our results for total precipitation to the monthly Level 3 gridded (0.5^o^ latitude by 0.5^o^ longitude) total precipitation data retrieved by the Tropical Rainfall Measuring Mission (TRMM) satellite (3A12, V7, downloaded from the NASA Giovanni website on March 27, 2017). The comparisons are shown in Figure 2. The WRF-Chem simulations do a reasonably good job of simulating the general distribution of precipitation in each season, but the model generally overestimated precipitation in each season, consistent with the findings of Kumar et al. (2015b). As noted by Kumar et al. (2015b), previous studies have also shown difficulty with accurately simulating monsoon rainfall over South Asia (e.g., Ratnam & Kumar, 2005; Rakesh et al., 2009; Kumar et al., 2012a). Kumar et al. (2012a) showed that their WRF simulated meteorology in this region was of sufficient quality for use in air quality simulations despite a similar overestimate of precipitation. Thus while this overestimate of precipitation is an important limitation on our conclusions in this work, it is unlikely to alter our general conclusions about the contributions of different sources and regions on the BC deposition to the HKHK region. However, it might significantly affect our estimates of the BC mass mixing ratio in snow (biasing that value low).

## 4 BC Deposition By Source

### 4.1 Current Climate

**Figure 3.**
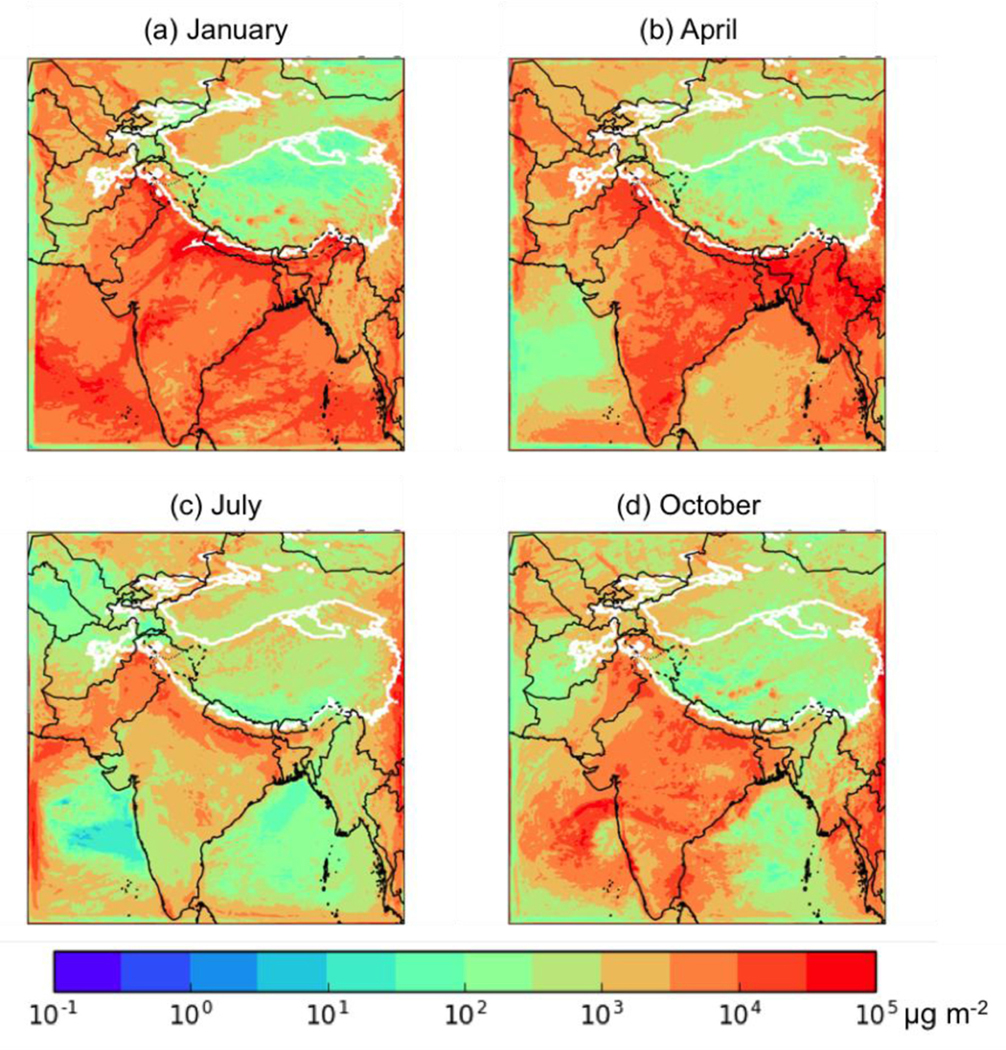
Total BC deposition (μg m^−2^) between the 15^th^ and 29^th^ of (a) January, (b) April, (c) July, and (d) October of 2013. Thick white contour shows the boundary of the 700 hPa surface pressure region.

Throughout this paper, “total BC deposition” refers to the sum of the wet and dry deposition fluxes of BC. Figure 3 shows the total BC deposition for the 14-day analysis period of each month simulated for 2013. As expected, these values are generally highest near high-emission regions and lower over the HKHK region. The BC deposition is higher in the western Karakoram and Hindu Kush ranges near the Pakistan-China and India-China borders, respectively, and lower over the Himalayas in the east. The seasonal cycle of deposition peaks in January over India, the Arabian Sea, and the Bay of Bengal, but the seasonal peak in deposition over the HKHK region varies, with the southern regions peaking in January to April and the northern regions peaking in July to October.

**Figure 4.**
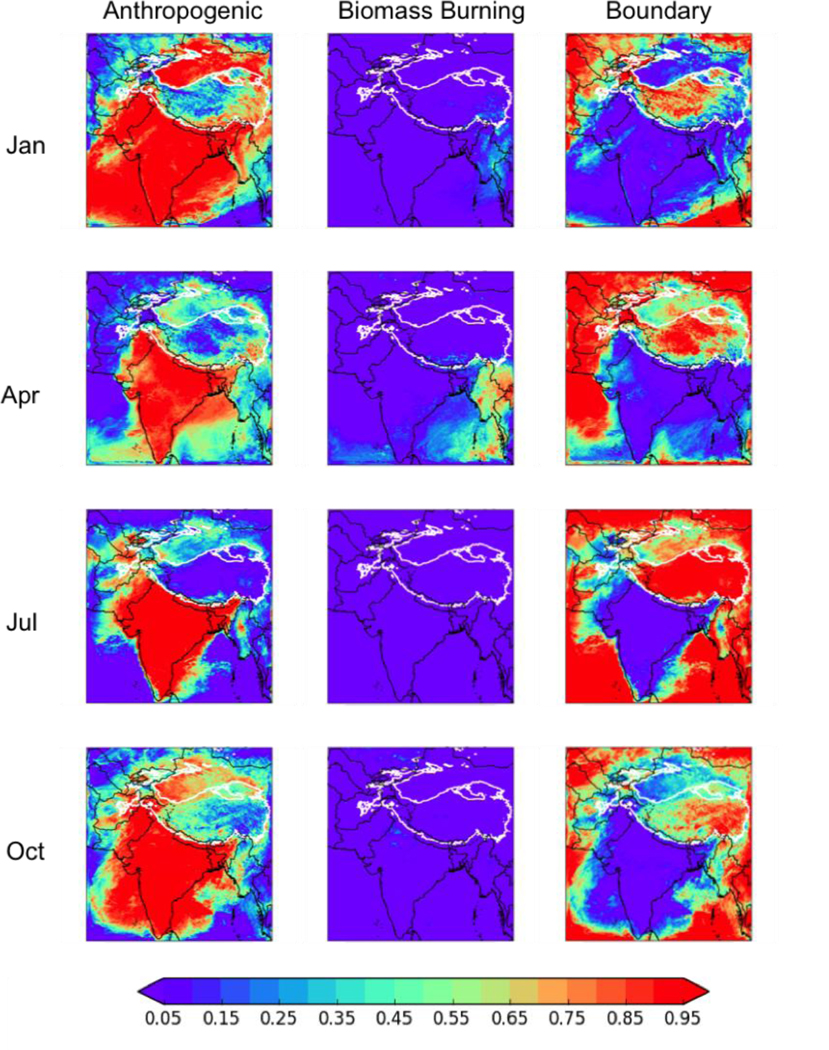
The fractions of the total BC deposition from (left to right) anthropogenic sources in the modeling domain, biomass burning (i.e., wildfires) in the modeling domain, and boundary conditions (i.e., all sources outside the domain) for the four months simulated in 2013. Thick white contour shows the boundary of the 700 hPa surface pressure region.

Figure 4 shows the division of this BC deposition between anthropogenic sources in the modeling domain, biomass burning (i.e., wildfires) in the modeling domain, and the boundary conditions (and thus from all sources outside the domain). Note that as the analysis period is after our 2-week spin-up, the fraction of deposition from the initial conditions is negligible everywhere. Anthropogenic sources are responsible for most of the deposition over and near India regardless of season, while biomass burning BC peaks over Myanmar and SE Asia in April of 2013. BC from sources outside of our WRF domain are consistently important in the northern part of the domain, including strong impacts in the HKHK regions peaking during the Summer Monsoon in July. This suggests that sources of BC outside of South Asia are responsible for a significant fraction of the BC deposition in these regions, likely from long-range transport of BC in the free troposphere that deposits to the mountains of the HKHK region. This is consistent with the global modeling results of Kopacz et al. (2011), who found that African biomass burning and Middle Eastern fossil fuel combustion can significantly contribute to the BC reaching the Himalayas and Tibetan Plateau. However, in this work we did not track the relative contributions of different global sources of BC to the tagged boundary condition BC (i.e., no tagged tracers were used in the MOZART-4/GEOS-5 or GISS-E2-R models used to generate the boundary conditions), nor did we track the contribution from the different boundaries separately, so we are unable to provide additional information on the relative contribution of different source regions or categories on the tagged boundary condition BC deposition. This could be explored in future work.

To be more quantitative in our analysis, the HKHK “mountain” regions were defined as any region with a surface pressure consistently above 700 hPa (see white contour in Figure 4), and thus well in the free troposphere. Note that this 700 hPa threshold is only used to provide summary statistics for mountainous regions in the domain, and that the fractional contributions from different sources can vary significantly within this region. The BC deposition from each tagged BC tracer over this region was then summed for all grid boxes with surface pressure consistently above 700 hPa for the 14 days in the evaluation period, and the ratios of these sums were used to define the average fractional contribution from each source. The fractional contributions of the different sources showed a strong seasonal cycle, with anthropogenic sources of BC in the domain accounting for 62% (113 Mg d^−1^ out of a total of 183 Mg d^−1^) of the HKHK deposition in January, 45% (111 Mg d^−1^ out of 249 Mg d^−1^) in April, and 54% in October (81.4 Mg d^−1^ out of 151 Mg d^−1^), but only 13% (25.7 Mg d^−1^ out of 195 Mg d^−1^) in July, when the contribution from sources outside the domain peaked at 87% (169 Mg d^−1^). Biomass burning inside the model domain was responsible for 3–4% of the BC deposition to the HKHK region in January (6.4 Mg d^−1^) and April (10.0 Mg d^−1^) and a negligible amount (<< 1%) in July (0.07 Mg d^−1^) and October (0.07 Mg d^−1^).

#### 4.1.1 Anthropogenic BC Deposition by Fuel Type

**Figure 5.**
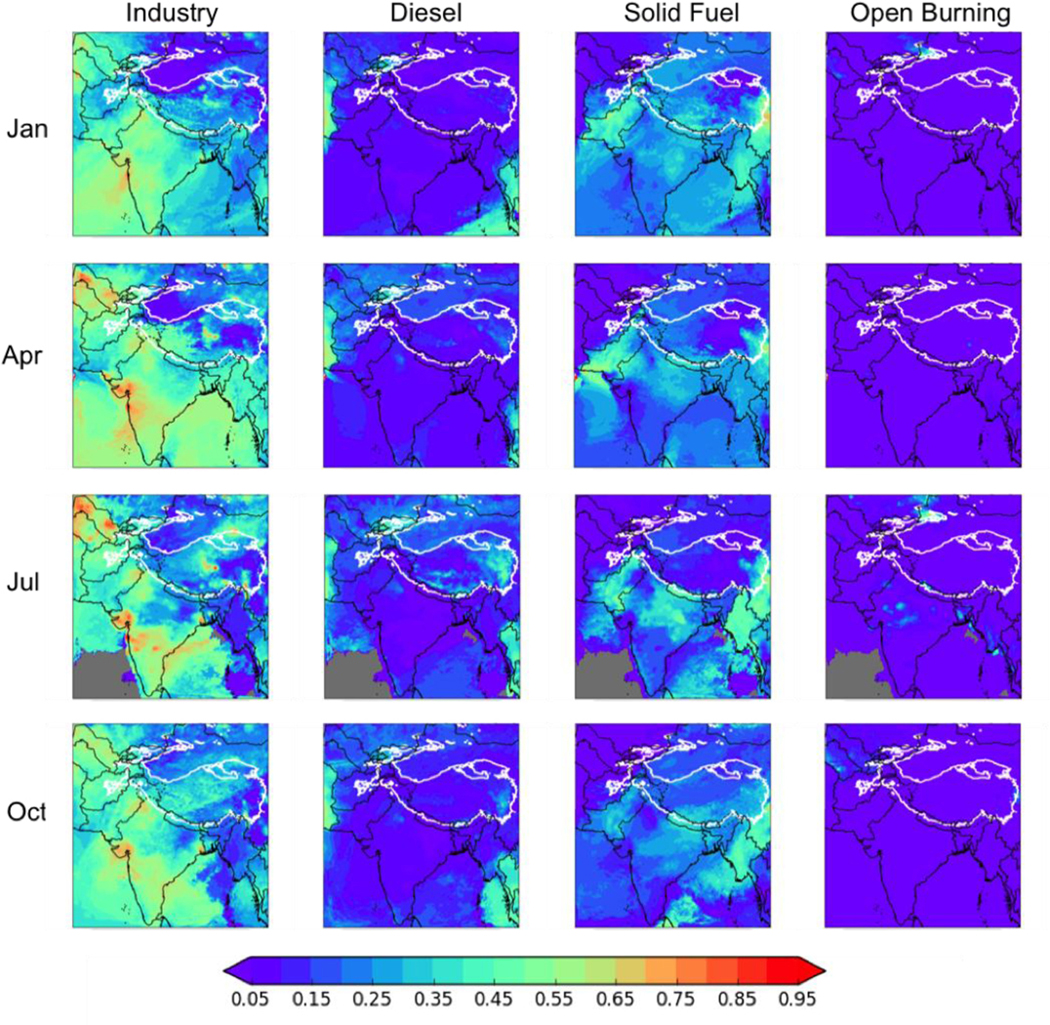
Fractions of the total *in-domain anthropogenic* deposition of BC from (left to right) industry (including brick kilns), diesel fuel, solid fuel, and open (agricultural) burning for 2013. Thick white contour shows the boundary of the 700 hPa surface pressure region. Gray areas are areas where the total in-domain anthropogenic deposition of BC is near zero and so a fraction cannot be calculated.

Figure 5 shows the fraction of the *in-domain anthropogenic* (not total) BC deposition from each of four major anthropogenic sources: industry (including brick kilns), diesel fuel, residential solid fuels, and open burning. Note that, as Figure 5 shows the fractional contribution to in-domain anthropogenic BC deposition, the results may not be meaningful near the boundaries of the simulation where boundary condition BC dominates the deposition. In the HKHK region, industry and solid fuel are again important in-domain anthropogenic sources, with industry contributing between 31–41% (minimum in July of 8.0 Mg d^−1^, maximum in April of 46.4 Mg d^−1^) and solid fuel 14–28% (minimum in July of 3.5 Mg d^−1^, maximum in January of 31.4 Mg d^−1^). Diesel fuel accounts for 7–10% of the HKHK BC deposition in January (8.1 Mg d^−1^), April (10.0 Mg d^−1^), and October (8.0 Mg d^−1^), but accounts for 18% in July (4.7 Mg d^−1^). The fractional contribution from open burning is less than 3% (maximum of 1.6 Mg d^−1^) year-round, with the fractional contribution peaking in July but the absolute value peaking in January. However, note that as in-domain anthropogenic sources account for between 13–62% of the total BC deposition in this region, industry and residential solid fuel generally account for less than 25% of the total BC deposition to the region.

#### 4.1.2 Anthropogenic BC Deposition by Region

The fraction of the *in-domain anthropogenic* (not total) deposition of BC that is due to anthropogenic sources in the six different countries studied are shown in the Supplemental Material for each season (Figures S1 to S4). As expected, each country has a strong influence on anthropogenic BC deposition within their own jurisdiction, but there is significant cross-boundary transport of BC in South Asia. For the HKHK region, Bangladesh and Myanmar generally have negligible impacts, while the other four nations (China, India, Nepal, and Pakistan) have noticeable contributions. The relative importance of the different nations also varies with season, with China and Pakistan contributing more to in-domain anthropogenic deposition in July, while India’s contribution peaks in October and January. However, note that as in-domain anthropogenic sources only account for between 13–62% of the total BC deposition in this region, the relative contributions of each nation to the total BC deposition in the HKHK region is generally well below 50%.

#### 4.1.3 Snowfall and Mass Fraction of BC in Snow

**Figure 6.**
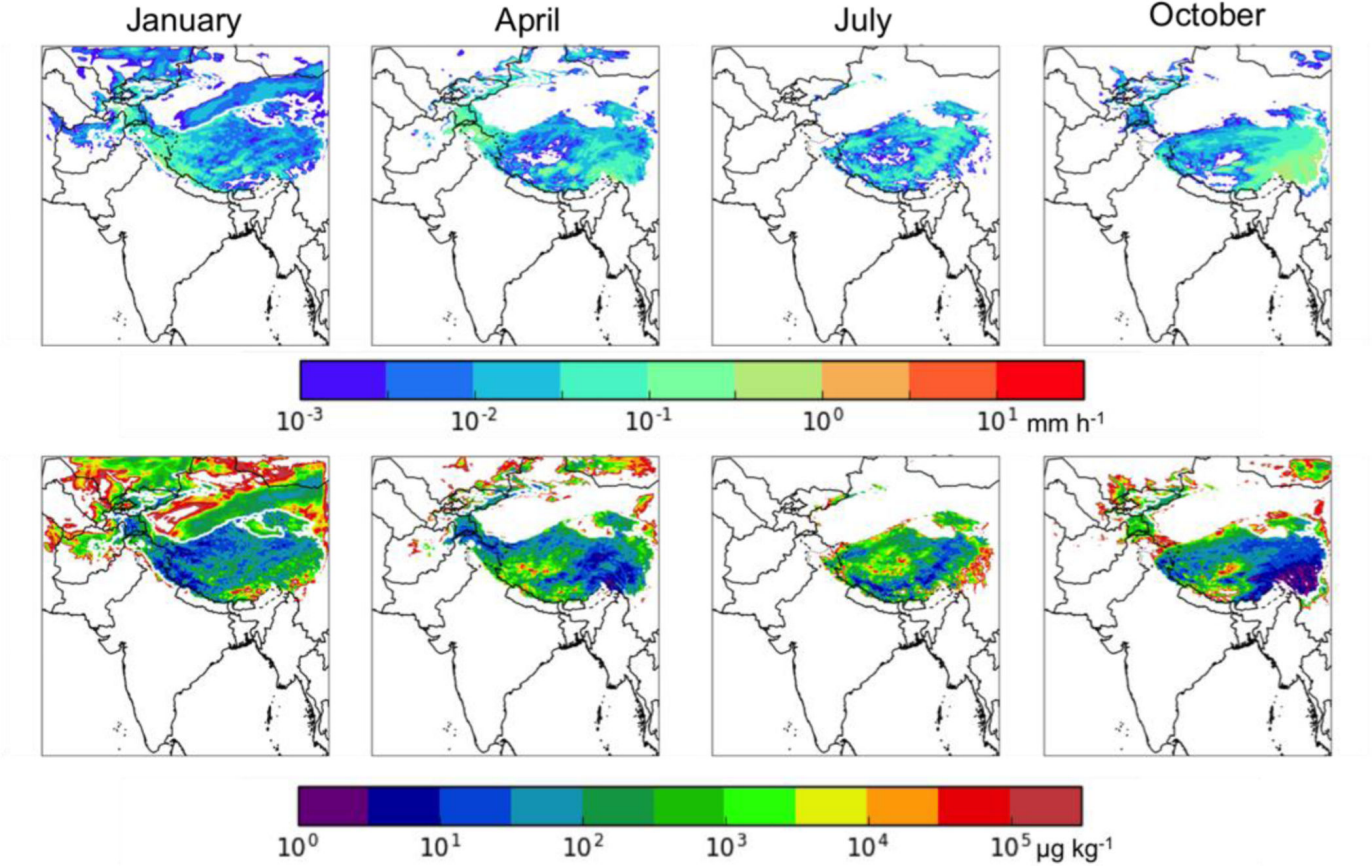
(top row) Average snowfall (mm h^−1^) during the analysis period of (left to right) January, April, July, and October 2013. (bottom row) Mass ratio of BC deposition to snowfall (μg BC/kg snow).

The top row of Figure 6 shows the average snowfall during the analysis period of each month in mm h^−1^. Snowfall is predicted to occur over the HKHK region year-round. The bottom row of Figure 6 shows the mass ratio of the total (wet and dry) deposited BC to the snowfall in μg BC (kg snow)^−1^. There is a large variation in this mixing ratio, with hot spot values > 10^4^ μg BC (kg snow)^−1^ in the HKHK region, leading to above-average impacts of BC on glacier albedos in these locations. The mass fraction of BC in fresh snow appears to reach a minimum in January (winter monsoon), due in part to the relative decrease in total BC deposition in the HKHK region in this period and in part to the relative increase in snowfall.

Table 5 compares the model-estimated mass mixing fraction of BC in snow with the observations used in the GEOS-Chem study of He et al. (2014). Like GEOS-Chem, the WRF-Chem model does not directly predict BC in snow at the surface. For this evaluation, we estimate BC concentration in snow in the model as the ratio of total BC deposition to total precipitation (Kopacz et al., 2011; Wang et al., 2011; He et al., 2014). Our discussion of the inherent uncertainties in this approach also follows that of He et al. (2014). The use of total precipitation may lead to an underestimate of the BC mixing ratio in snow – Bonasoni et al. (2010) found that precipitation can be partly in the form of rain even at altitudes of 5 km in the Himalayas. Other biases and uncertainties in the modeled precipitation field would also affect the model calculation of BC concentration in snow.

There are other important limitations to this approach. We implicitly assume a well-mixed layer of BC and snow, and that the sampling period of the observations corresponds to similar conditions as the two weeks simulated here. However, BC content is not uniform throughout the snow column, and thus there may be errors since the depth of the snow column sampled in the observations may not correspond to the two-week snow layer simulated by the model. In fact, the results for certain combinations of months and sites with low modeled levels of precipitation give much higher values of the BC mixing ratio (»10^3^ μg kg^−1^) than seen in the observations in part due to the neglect of this depth dependence. We attempt to mitigate this by computing an average of our four two-week periods by summing both the deposition of BC and the total precipitation for all four periods and then taking the ratio of the two summed quantities. This average model value showed a better correlation with the observed values than choosing the two-week period closest in season with the observations. This is reported as the mean value in Table 5, but the individual two-week ratios are also reported.

Our approach also neglects the aging of surface snow and the internal mixing of snow and BC. As noted by He et al. (2014), this may be an especially important issue for comparisons in the central Tibetan plateau and to the north of the plateau, where snowmelt has been suggested to strongly increase BC concentration in snow (Zhou et al., 2007; Ming et al., 2013).

Using the mean model values as our model estimates, the model has a mean normalized bias (MNB) of −11% and a mean absolute normalized gross error (MANGE) of 52%. The correlation between the modeled and observed values is relatively low (linear correlation coefficient *R* of 0.49), even though the regression slope is close to 1 (1.15). We feel this performance is sufficient to allow the model to be used to examine the contribution of different source types and regions to the total BC deposition rate. However, while the observations show a reasonably strong inverse correlation with altitude (*R* of −0.66), the modeled estimates show little correlation with altitude (*R* of −0.1), suggesting the model may not be fully resolving the effects of topography in the region.

As noted above, the model performance is worse if we instead use the closest corresponding month for the model estimate (MNB of 52%, MANGE of 194%, *R* of 0.3). In addition, as noted above some of the two-week values are »10^3^ μg kg^−1^ due to low total precipitation estimates for the period, although the highest two-week value corresponding to an observation is the October estimate for the Haxilegen River north of the Tibetan Plateau (978 μg kg^−1^). Most of the seasonal variation in the modeled estimates for each site is due to seasonal variation in the total precipitation. In the model, the total precipitation varies by 1–3 orders of magnitude between the seasonal minimum and maximum depending on the site, while the corresponding variation of total BC deposition (wet plus dry) is between a factor of 2 to 30. Thus model errors in the seasonal cycle of total precipitation likely dominate the uncertainty in the BC mixing ratio in snow, with the uncertainties in the BC deposition rates having less of an effect.

### 4.2 Future Climate: No Further Control versus Mitigation Scenario

This section compares the BC deposition results for the two different future emission scenarios evaluated in this work – the NFC and MIT cases (Section 2.2). As the relative changes between the two scenarios are similar regardless of the phase of ENSO (Section 4.3), our discussion here focuses on the “moderate” ENSO case.

Table 6 shows the BC surface concentrations for the NFC and MIT cases at the sites used in the evaluation of Kumar et al. (2015a). The concentrations for the NFC cases are not significantly different from those for current conditions for areas dominated by local emissions, such as Dehli, Kanpur, and Kharagpur. However, concentrations in the sites at high elevations in the HKHK region are generally reduced, likely reflecting reductions in BC emissions in the rest of the world under RCP 4.5. However, under the MIT scenario surface BC concentrations are dramatically reduced relative to the NFC scenario by factors of 2–3 or more at all of the surface stations examined except for Minicoy in January and April. This shows that the mitigation efforts included in the MIT scenario would significantly reduce surface BC concentrations over South Asia.

**Figure 7.**
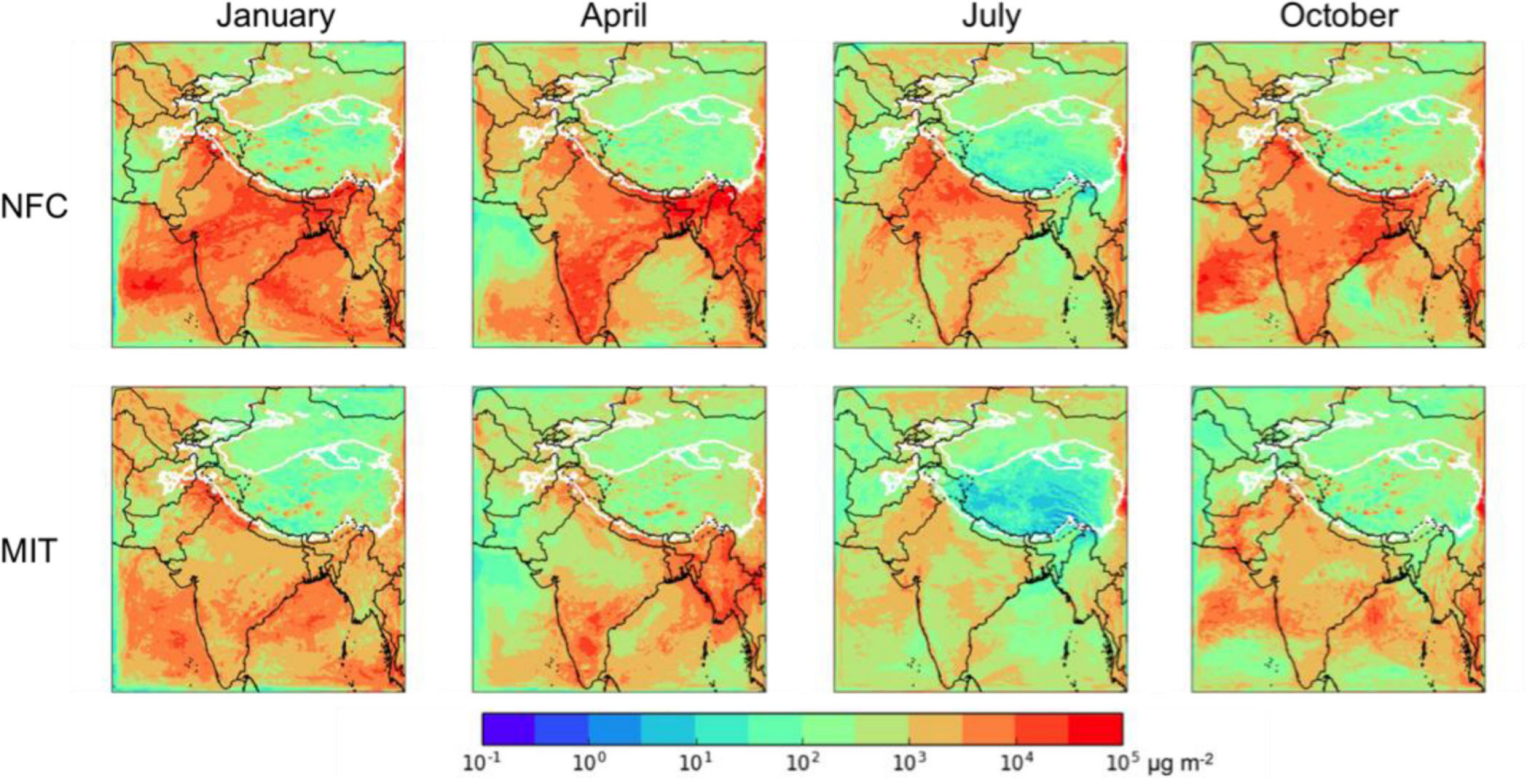
Total BC deposition (μg m^−2^) for the no further control (NFC, top row) and mitigation (MIT, bottom row) 2050 emission scenarios for a moderate ENSO year in (left to right) January, April, July, and October. Thick white contour shows the boundary of the 700 hPa surface pressure region.

Figure 7 shows the total deposition of BC in each season for the NFC (top row) and MIT (bottom row) scenarios. The seasonal cycle is similar in both cases, and is similar to that for the 2013 simulations (Section 4.1). Again, the NFC future case is generally similar to the current conditions over South Asia, as expected, but the total BC deposition to the HKHK region averaged over the seasons is 33% lower than in 2013 (130 Mg d^−1^ versus 195 Mg d^−1^). Total BC deposition is clearly lower in the MIT case (relative to the NFC case) over India, Pakistan, the Arabian Sea and Bay of Bengal relative to the NFC case. However, the changes are smaller over much of the HKHK region (55% below 2013 levels averaged over the seasons, or 87.3 Mg d^−1^), with the exception of the month of July, which has clearly lower BC deposition in the HKHK region in the MIT scenario.

**Figure 8.**
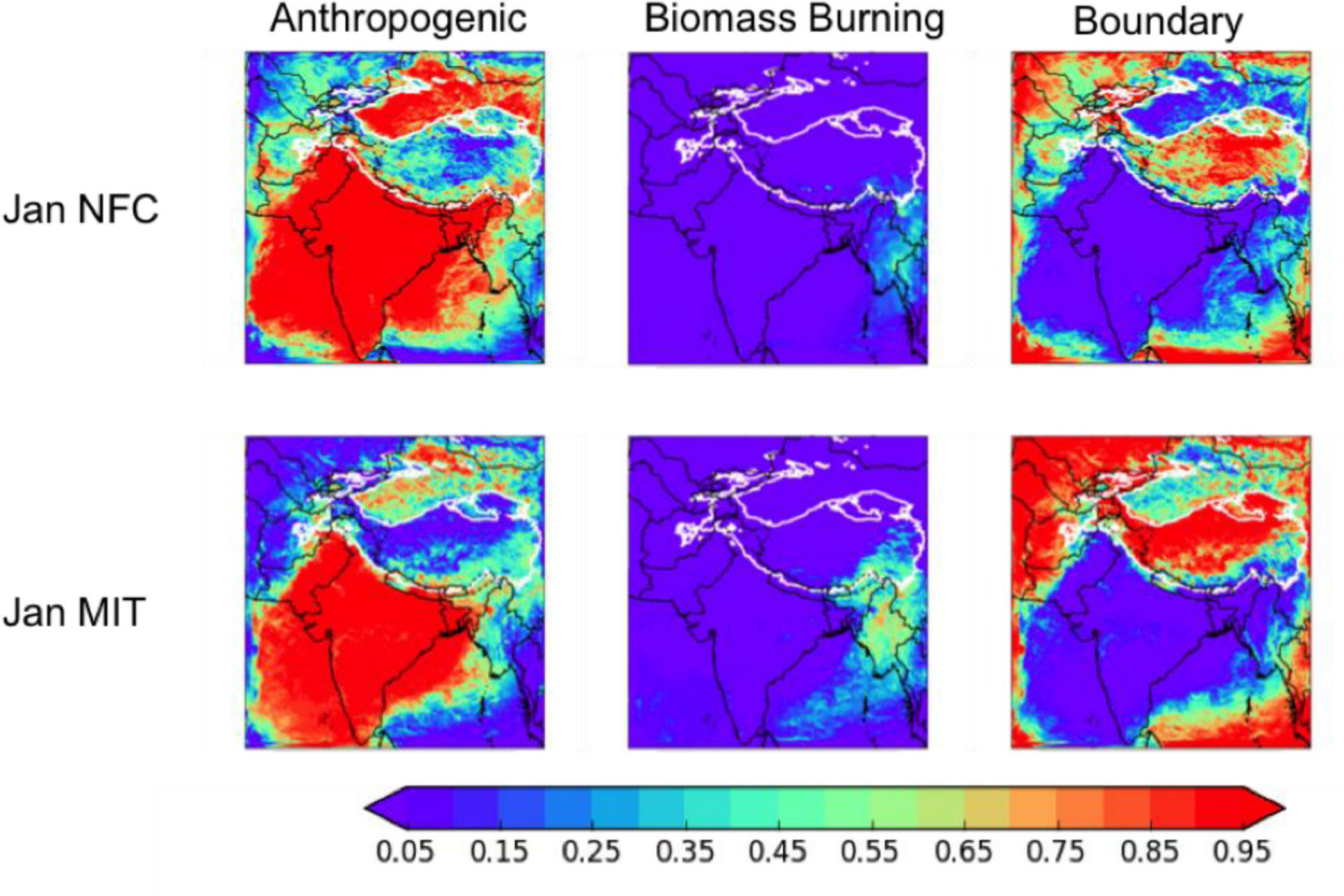
The fractions of the total BC deposition from (left to right) anthropogenic sources in the modeling domain, biomass burning in the modeling domain, boundary conditions (i.e., all sources outside the domain) in January of the moderate ENSO future cases with two emission scenarios for 2050 - no further control (NFC, top row) and mitigation (MIT, bottom row). Thick white contour shows the boundary of the 700 hPa surface pressure region.

Figure 8 shows the fractional contribution of in-domain anthropogenic sources in the modeling domain, biomass burning in the modeling domain, and the boundary conditions (and thus from all sources outside the domain) to the total BC deposition in January in the NFC and MIT scenarios – the choice of January is arbitrary, as the conclusions of our analysis are valid for all four seasons. In general, the fractional contribution from in-domain anthropogenic sources is lower in the MIT case in the HKHK region – 15–31% (4.7 to 40.4 Mg d^−1^) depending on month, as opposed to 45–56% (46.1 to 85.9 Mg d^−1^) in the NFC case – indicating out of domain sources dominate the BC deposition in the HKHK region under the MIT emissions scenario.

**Figure 9.**
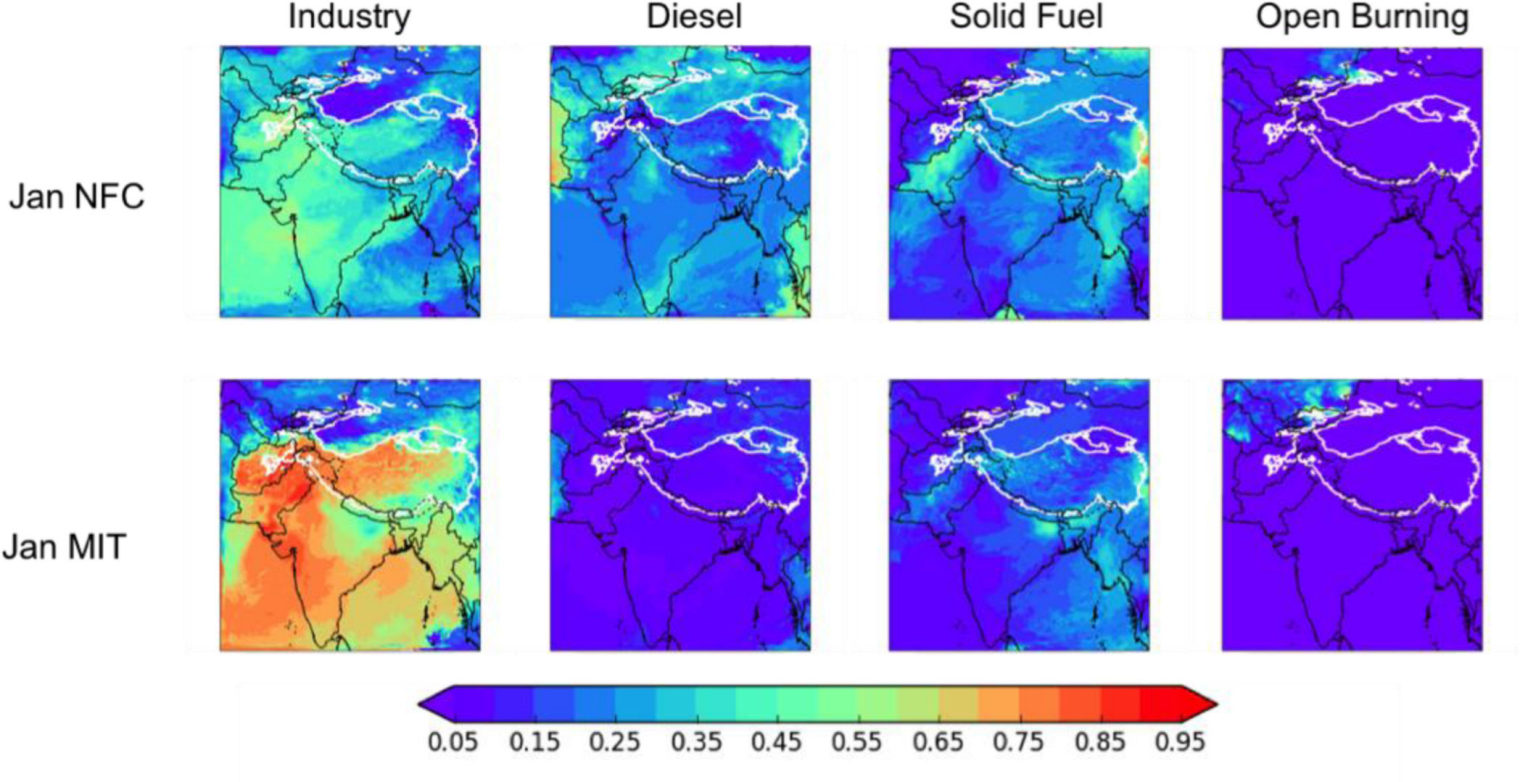
Fractions of the total *in-domain anthropogenic* deposition of BC from (left to right) industry (including brick kilns), diesel fuel, solid fuel, and open burning for January of the moderate ENSO future case using two emission scenarios for 2050 - no further control (NFC, top row) and mitigation (MIT, bottom row). Thick white contour shows the boundary of the 700 hPa surface pressure region.

Figure 9 shows the breakdown of the anthropogenic deposition by source type for January (again, the conclusions of our analysis are independent of season). In the MIT scenario, the fractional contribution of on-road diesel engines dramatically decreases across the domain relative to the NFC scenario, while the fractional contribution from industry increases. The fractional contributions of residential solid fuel burning and open agricultural burning do not change much, but as the overall anthropogenic deposition is less in the MIT scenario than in the NFC scenario, absolute BC deposition from these sectors is lower in the MIT scenario as well. Over the HKHK region, the diesel contribution again changes dramatically between the MIT (5–11%, or 0.5 to 2.3 Mg d^−1^) and NFC (18–26%, or 12.1 to 15.4 Mg d^−1^) scenarios, with open burning making only a negligible contribution (<4%) in all scenarios. Industry (including brick kilns) accounts for 47–70% (2.1 to 28.5 Mg d^−1^) of the in-domain anthropogenic BC deposition in the HKHK region under the MIT scenario, with residential solid fuel only contributing 12–21% (0.6 to 6.0 Mg d^−1^) However, under the NFC scenario, industry contributes only 33–42% (16.4 to 22.7 Mg d^−1^) of the in-domain anthropogenic BC deposition in the HKHK region, while solid fuel burning contributes 20–27% (9.1 to 23.4 Mg d^−1^).

**Figure 10.**
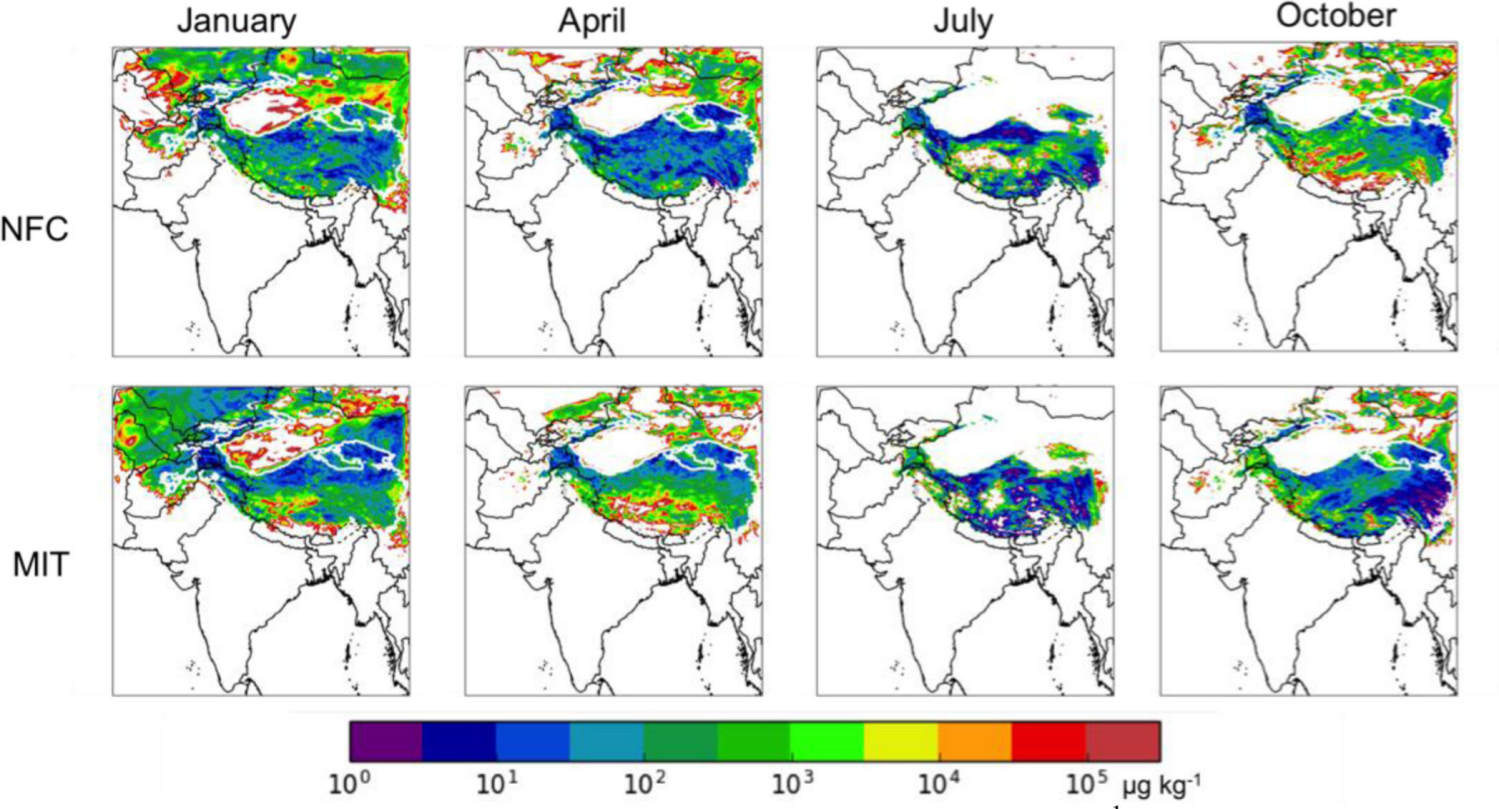
Mass ratio of BC deposition to snowfall in μg BC (kg snow)^−1^ for the no further control (NFC, top row) and mitigation (MIT, bottom row) emission scenarios for 2050 for a moderate ENSO year in (left to right) January, April, July, and October.

Figure 10 shows the mass ratio of the deposited BC to the snowfall for both the NFC and MIT scenarios. The overall patterns are similar, as out of domain sources dominate the HKHK contributions and the emissions from out of domain sources are the same in the two scenarios simulated here, but there are significant differences near the Nepal/China border in January and April, with much larger mass fractions present in the MIT scenario. These differences in the mixing ratio of BC in the snow may be due to a reduction in snowfall in this area in the MIT case relative to the NFC case. The snowfall in the HKHK region is 21% higher in January and 206% higher in April in the NFC case relative to the MIT case, but as the total BC deposition in these regions is also higher in the NFC case, these localized areas near the Nepal/China must have some combination of lower BC deposition and higher snowfall in the NFC case, which is balanced out by higher NFC deposition in much of the HKHK region.

### 4.3 Future Climate: El Niño versus La Niña

This section compares the BC deposition results for the three different phases of ENSO evaluated in this work. Our discussion here focuses on the differences when the no further control (NFC) scenario is used, but the results are similar for the MIT case. Table 7 shows the BC surface concentrations at the sites used in the evaluation of Kumar et al. (2015a) for the three phases of ENSO using the NFC emission scenario. For regions dominated by local emissions, there is little change with ENSO phase. However, coastal sites such as Minicoy and Port-Blair are very sensitive to the changes in ENSO and associated changes in the meteorology, as the amount of BC transported to these sites depends on large-scale transport patterns in the region. The elevated sites in the HKHK region also show variability with the three ENSO phases simulated here that can be a factor of 2–3 higher. However, it is unclear if these inter-annual changes correlate with ENSO itself, or if they reflect other modes of inter-annual variability.

**Figure 11.**
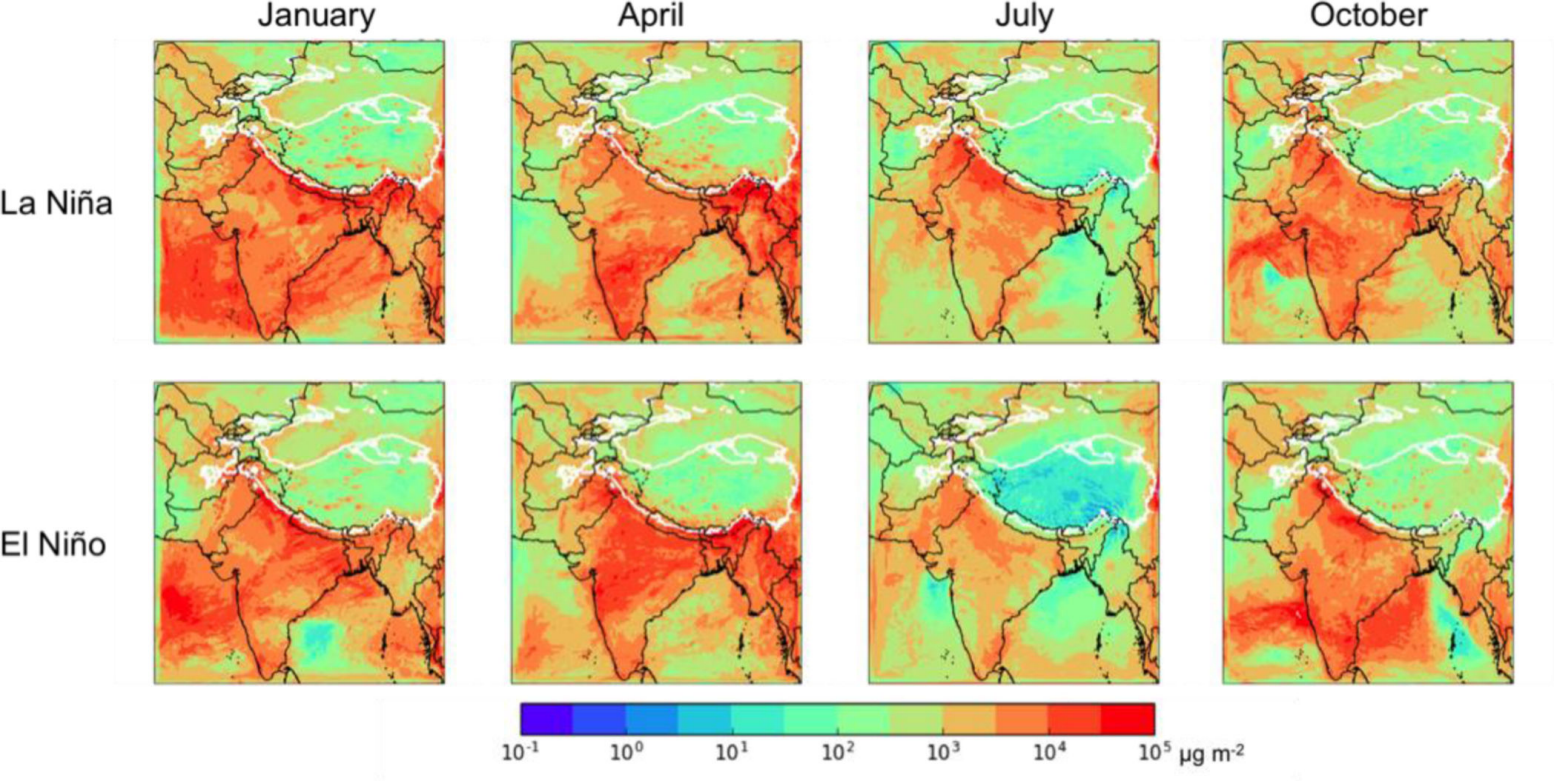
Total BC deposition (μg m^−2^) for the no further control (NFC) emission scenario for 2050 for a La Niña year (top row) and an El Niño year (bottom row) in (left to right) January, April, July, and October. Thick white contour shows the boundary of the 700 hPa surface pressure region.

Figure 11 shows the total deposition of BC in each season for the La Niña (top row) and El Niño (bottom row) cases, while Table 8 summarizes the average total BC deposition for each month and each phase of ENSO under the NFC scenario, as well as the contributions of in-domain anthropogenic sources and sources outside of South Asia (BDY) on this deposition. Total BC deposition during the rainy season (July) is lower in the El Niño year (58 Mg d^−1^) than in the La Niña and moderate years (96 and 84 Mg d^−1^, respectively), while October deposition is lower in the La Nina year (117 Mg d^−1^) than in the El Niño and moderate years (135 and 137 Mg d^−1^, respectively). Interestingly, the seasonally averaged deposition is higher in the La Niña and El Niño years (173 and 169 Mg d^−1^, respectively) than in the moderate ENSO phase (130 Mg d^−1^). This appears to be due to higher deposition rates in January and April during La Niña and El Niño phases than in the moderate ENSO year, with the increased deposition mainly coming from in-domain anthropogenic sources. Contributions from the boundary conditions have a different seasonal pattern in the El Niño year than in the other years (with El Niño year having relatively more deposition from these sources in April and relatively lower in October) but the seasonal averages are similar. This suggests that the main effect of ENSO is to increase deposition from in-domain anthropogenic sources during the dry season (January) and the following transition to the wet season (April). However, it is again unclear if these inter-annual changes are due to ENSO itself, or if they reflect other modes of inter-annual variability.

**Figure 12.**
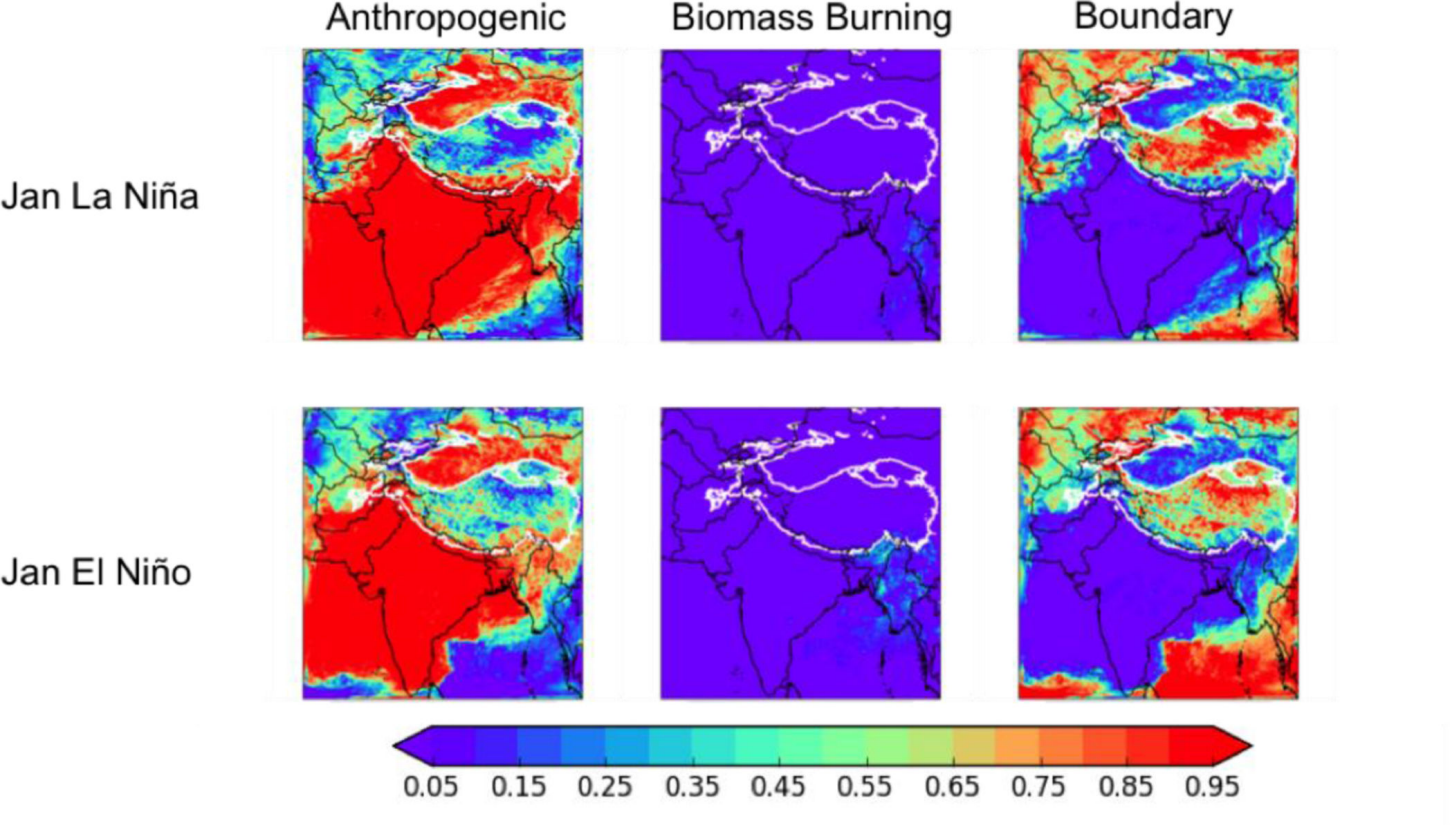
The fractions of the total BC deposition from (left to right) anthropogenic sources in the modeling domain, biomass burning in the modeling domain, boundary conditions (i.e., all sources outside the domain) in January of the La Niña (top row) and El Niño (bottom row) future cases with the no further control (NFC) emission scenario for 2050. Thick white contour shows the boundary of the 700 hPa surface pressure region.

**Figure 13.**
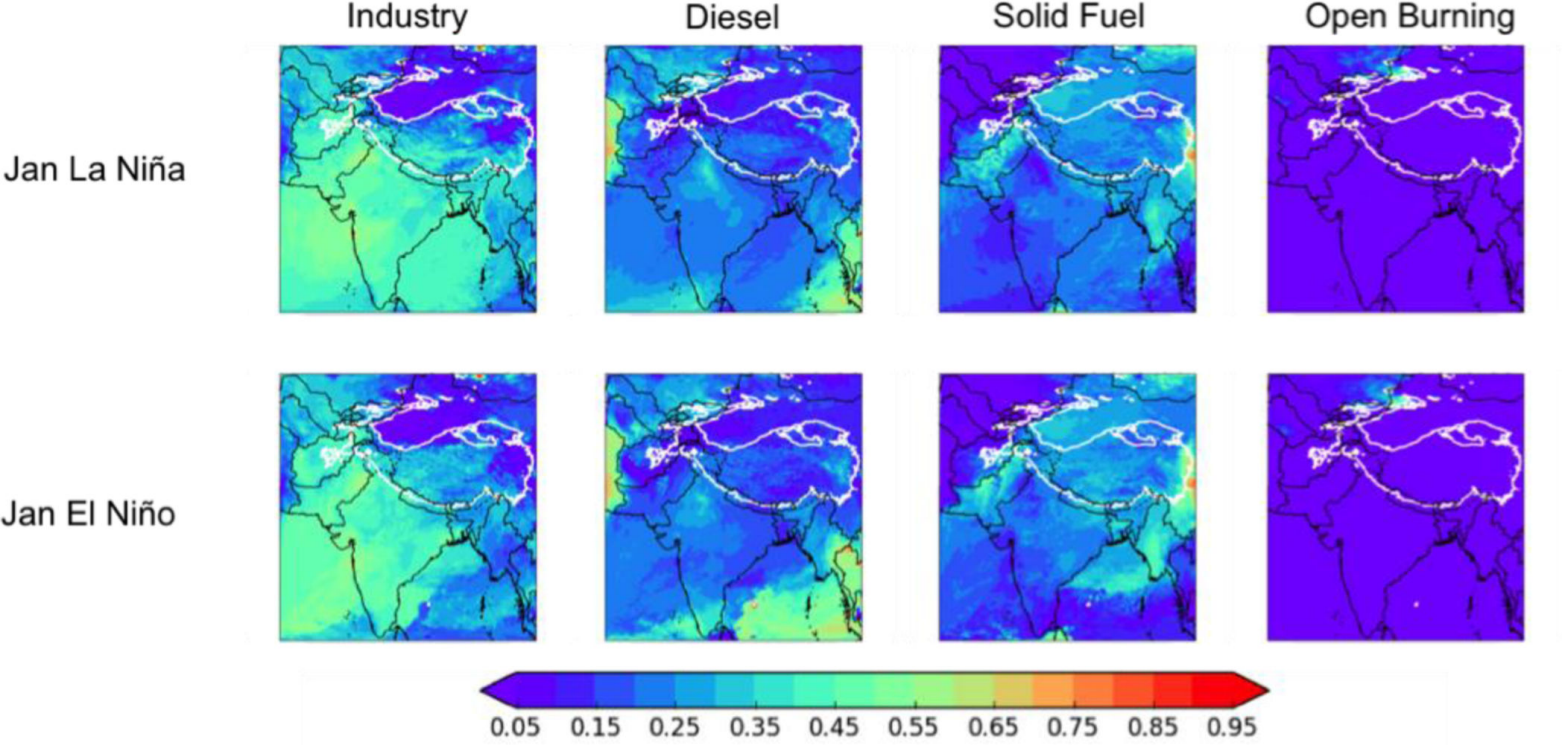
Fractions of the total *in-domain anthropogenic* deposition of BC from (left to right) industry (including brick kilns), diesel fuel, solid fuel, and open burning in January of the La Niña (top row) and El Niño (bottom row) future cases with the no further control (NFC) emission scenario for 2050. Thick white contour shows the boundary of the 700 hPa surface pressure region.

Figure 12 shows the fractional contribution of in-domain anthropogenic sources in the modeling domain, biomass burning in the modeling domain, and the boundary conditions (and thus from all sources outside the domain) to the total BC deposition in January for the La Niña (top row) and El Niño (bottom row) cases under the NFC emission scenario (again, the conclusions of our analysis are independent of season). In the moderate ENSO year, the in-domain anthropogenic fractional contribution varied between 32% in January to 55% in July. In the La Niña year, the fractional contribution from in-domain anthropogenic sources was fairly steady, varying between 62% in January and 50% in October. In the El Nino year, the fractional contribution from in-domain anthropogenic sources varied between 39% in July and 66% in January. Figure 13 shows that the relative contributions of different anthropogenic sources to the total in-domain anthropogenic deposition is relatively independent of ENSO – for example, the industrial contributions is between 32–42% for all three years..

**Figure 14.**
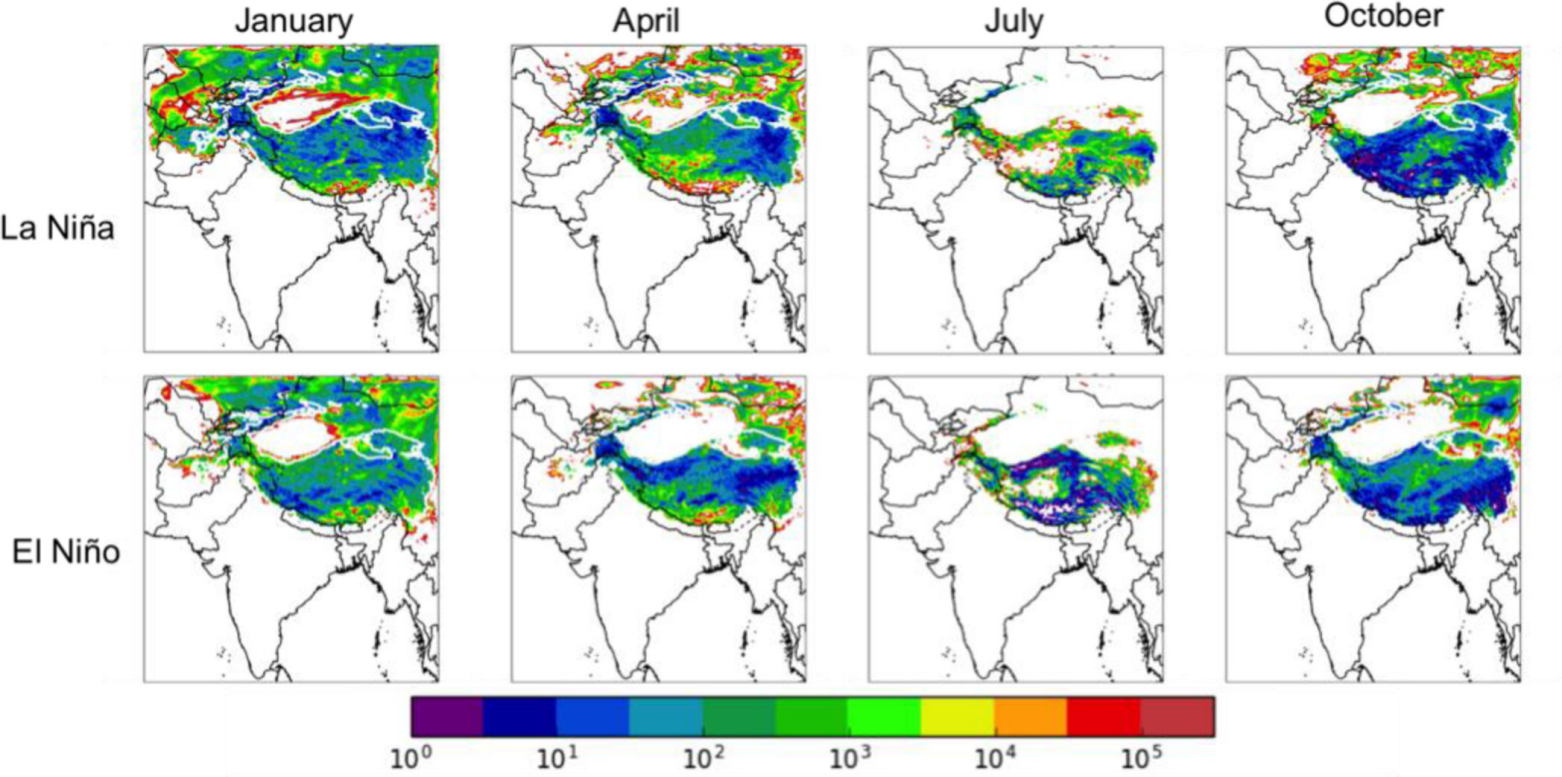
Mass ratio of BC deposition to snowfall (μg BC/kg snow) for the La Niña (top row) and El Niño (bottom row) future cases with the no further control (NFC) emission scenario for 2050 in (left to right) January, April, July, and October. Thick white contour shows the boundary of the 700 hPa surface pressure region.

Figure 14 shows the mass ratio of the deposited BC to the snowfall for the La Niña (top row) and El Niño (bottom row) cases under the NFC emission scenario. Again the overall patterns are similar regardless of ENSO phase, but the locations of specific hot spots, likely caused by low simulated snowfall in these regions, can vary significantly between the phases. However, as with the BC deposition results, it is unclear if these inter-annual changes in the mass fraction of BC in fresh snow correlate with ENSO itself, or if they reflect other modes of inter-annual variability.

## 5. Conclusions

WRF-Chem v3.6.1 with tagged BC tracers and a modified version of the ECLIPSE 5a emission inventory – including the improved residential emission inventory of Winijkul & Bond (2016) and Winijkul et al. (2016), and where the ratio between the ECLIPSE 5a CLE projection for 2050 and the RCP 4.5 projection was used to scale the ECLIPSE 5a NFC and MIT scenarios for 2050 – were used to investigate the sources impacting BC deposition to South Asia in general and the HKHK region in particular (defined here as regions in South Asia with surface pressures consistently below 700 hPa). Simulations were performed both under current conditions and in the 2040–2050 period using two emission scenarios and in three different phases of ENSO. The future cases used meteorological and boundary conditions from the GISS-E2-R model with prognostic aerosols and aerosol-cloud microphysics included and driven by emissions from RCP 4.5 and RCP 4.5 except South Asian emissions mitigation.

While our modelling configuration is able to simulate the atmospheric concentrations and wet and dry deposition fluxes of BC, it does have some inherent limitations. For example, wet deposition of BC is calculated using a grid-scale parameterization that only accounts for in-cloud scavenging, not below cloud or impaction scavenging, and thus could underestimate the wet deposition of BC. The model also tends to overestimate the monsoon precipitation in South Asia and has difficulty reproducing the observed seasonal cycle of surface BC concentrations at Himalayan sites (predicting a January maximum whereas observations show an April maximum), similar to previous studies with WRF-Chem. We also only examined one RCP scenario, and thus our conclusions only apply to that particular scenario. Furthermore, given the uncertainties in the emission inventories, our estimates of the percentage contributions from the different source types and regions are likely uncertain to a factor of 2.

We find that under current conditions (i.e., the year 2013, a moderate ENSO year) sources from outside our South Asian modelling domain have a similar impact on total BC deposition to the HKHK region (35–87%, varying with month) as South Asian anthropogenic sources (13–62%), with the boundary contribution peaking in July. The in-domain anthropogenic contribution is from industry (primarily brick kilns) and residential solid fuel burning, which, when combined, account for 45–66% of the in-domain anthropogenic BC deposition to the HKHK region, with on-road diesel fuels making a smaller contribution (7–18%, peaking in July) and open burning accounting for less than 3% in all seasons. Other anthropogenic combustion sources of BC (e.g., waste burning) were not explicitly tracked but account for the remaining fraction of the in domain emissions.

Assuming the modified no further control (NFC) emission scenario, the total BC deposition to the HKHK region averaged over the seasons is 33% lower than in 2013. The contributions to BC deposition in the HKHK region in the NFC scenario are roughly evenly divided between in-domain anthropogenic sources (45–65%, depending on month and ENSO phase) and sources outside the domain (26–52%). The in-domain anthropogenic BC deposition has significant contributions mainly from industry (32–42%), solid fuel burning (17–28%), and diesel fuel burning (17–27%), with open burning again having a small (<4%) contribution.

Under the modified mitigation (MIT) emission scenario, the overall deposition decreases by 55% from 2013 levels, and the relative contribution from South Asian anthropogenic sources is significantly reduced to 11–34%, with sources outside the domain becoming relatively more important to the remaining deposition. Furthermore, the relative contribution of diesel fuel to the remaining in-domain anthropogenic deposition is dramatically reduced to 5–12%, while the relative contribution from industry increases to 42–70% and the relative contribution from solid fuel burning is steady at 9–25%.

We also examined how these 2040–2050 results are affected by different phases of ENSO. These changes are generally smaller than those between the NFC and MIT cases. The results suggest that the main effect of ENSO is to increase deposition from in-domain anthropogenic sources during the dry season (January) and the following transition to the wet season (April). However, these differences may be due more to general inter-annual variability in transport patterns than any specific changes associated with the ENSO mode.

In future work, we will use the high-resolution deposition maps developed in this project to determine the impact of different sources of BC on the albedos of glaciers in the HKHK region, and how these albedo changes affect glacier melt and water availability in the region. A better understanding of the sources of BC depositing on glaciers in this region, both today and in future climate projections, will help policy makers, civil society, and other stakeholders in South Asia understand which local actions may be taken to reduce or offset these and other potential impacts on public health, power availability, water resources and food security.

## Supplementary Material

1

## Figures and Tables

**Table 1.** Descriptions of simulations performed in this study

Simulation	Year^*^	ENSO Phase	Anthro. Emis. Year	Anthro. Emis. Scenario	Biomass Burn. Emis. Year
1	2013	Moderate	2010	Base	2013
2	2043	Moderate	2050	NFC	2013
3	2043	Moderate	2050	MIT	2013
4	2044	La Niña	2050	NFC	2011
5	2044	La Niña	2050	MIT	2011
6	2048	El Niño	2050	NFC	2015
7	2048	El Niño	2050	MIT	2015

* Note that the future years may not all come from the same initialization of the GISS-E2-R model.

**Table 2.** Parameterizations used for selected processes in WRF-Chem.

Process	Parameterization
Cloud microphysics	Morrison double moment (Morrison et al., 2009)
Radiation	RRTMG (Iacono et al., 2008; Mlawer et al., 1997)
Surface layer	MM5 Similarity Scheme (Beljaars, 1994)
Land surface model	Noah land surface (Tewari et al., 2004)
Planetary boundary layer	Yonsei university scheme (Hong et al., 2006)
Cumulus parameterization	Grell-3-D (Grell & Devenyi, 2002)
Dry deposition	Wesely (Wesely, 1989)
Wet deposition	Neu and Prather (Neu & Prather, 2012)
Dust emissions	In-line AFWA (Adams-Selin, 2012)
Aerosol scheme	GOCART (Chin et al., 2002)
Gas-phase chemistry	Not used
Photolysis	Not used
Biogenic emissions	Not used

**Table 3.** WRF-Chem simulated BC surface mass concentration (mean +/− standard deviation, μg m^−3^) averaged over 1 April to 28 April 2013, and compared with the observations and simulations of Kumar et al. (2015a). Sites within the HKHK region are highlighted in bold.

Site Name	Lat, long, alt	Observed (Mar-May)	Kumar et al. (2015a) (18 Mar-11 May 2006)	This work (1–28 Apr, 2013)	Ref.
Dehli	28.6°N, 77.2°E, 260 m	8–12	6.7±4.0	12.5±9.7	Beegum et al. (2009)
Kanpur	26.4°N, 80.3°E, 142 m	2–5	4.7±2.7	4.7±2.3	Ram et al. (2010)
Kharagpur	22.5°N, 87.5°E, 28 m	2–5	3.7±2.8	5.1±2.9	Beegum et al. (2009)
Dibrugarh	27.3°N, 94.6°E, 111 m	5–10	3.7±3.1	1.6±1.7	Pathak et al. (2010)
Trivandrum	8.5°N, 76.9°E, 3 m	1.8–3	0.9±0.6	1.0±1.0	Beegum et al. (2009)
Minicoy	8.3°N, 73.0°E, 1 m	0.065–0.22	0.24±0.15	0.07±0.26	Beegum et al. (2009)
Port-Blair	11.5°N, 92.7°E, 60m	1.3–1.8	0.7±0.8	0.06±0.10	Beegum et al. (2009)
**Nainital**	**29.4°N, 79.5°E, 1958 m**	**0.8–1.5**	**1.2±0.8**	**1.6±0.9**	Beegum et al. (2009)
**Nagarkot**	**27.7°N, 85.5°E, 2150 m**	**1.5**	**1.3±1.1**	**2.6±1.9**	Carrico et al. (2003)
**Lhasa**	**29.7°N, 91.1°E, 3663 m**	**2–3**	**0.42±0.25**	**0.26±0.22**	Zhang et al. (2008)
**Langtang**	**28.1°N, 85.6°E, 3920 m**	**0.5**	**0.8±0.5**	**0.70±0.66**	Carrico et al. (2003)
**NCO-P**	**28.0°N, 86.8°E, 5079 m**	**0.2–0.4**	**0.46±0.39**	**0.17±0.20**	Bonasoni et al. (2010)

**Table 4.** WRF-Chem simulated BC surface mass concentration (mean +/− standard deviation, μg m^−3^) averaged over the four months simulated in 2013. Sites within the HKHK region are highlighted in bold. Monthly average observed values (as reported by Kumar et al., 2015b) are presented where available.

Site Name	January 1–28 (Winter Monsoon)		April 1–28 (Monsoon Transition)		July 1–28 (Summer Monsoon)		October 1–28 (Monson Transition)	
	This Work	Obs.	This Work	Obs.	This Work	Obs.	This Work	Obs.
Dehli	18.1±8.5	10.9	12.5±9.7	4.2	4.6±4.2	2.7	12.7±9.7	6.5
Kanpur	9.5±2.9	10.9	4.7±2.3	6.3	1.7±1.7	1.8	3.9±2.9	4.9
Kharagpur	11.9±5.4	12.0	5.1±2.9	2.9	2.1±2.2	2.2	3.4±3.1	5.9
Dibrugarh	3.1±1.4	18.6	1.6±1.7	7.0	0.9±0.6	3.0	1.0±1.2	7.0
Trivandrum	2.8±1.5	6.1	1.0±1.0	3.1	1.5±1.4	1.9	1.1±1.0	3.0
Minicoy	0.30±0.73	0.92	0.07±0.26	0.23	0.005±0.005	0.10	0.01±0.01	0.22
Port-Blair	0.30±0.46	2.5	0.06±0.10	2.3	0.003±0.004	0.5	0.003±0.004	2.1
**Nainital**	**2.6±1.4**	**0.91**	**1.6±0.9**	**1.52**	**0.51±0.46**	**0.55**	**1.8±0.6**	**1.02**
**Nagarkot**	**3.5±1.7**	**N/A**	**2.6±1.9**	**N/A**	**0.75±0.58**	**N/A**	**1.2±1.0**	**N/A**
**Lhasa**	**0.37±0.21**	**N/A**	**0.26±0.22**	**N/A**	**0.05±0.05**	**N/A**	**0.24±0.19**	**N/A**
**Langtang**	**0.84±0.46**	**N/A**	**0.70±0.66**	**N/A**	**0.16±0.11**	**N/A**	**0.36±0.28**	**N/A**
**NCO-P**	**0.25±0.16**	**0.16**	**0.17±0.20**	**0.43**	**0.04±0.01**	**0.03**	**0.08±0.06**	**0.13**

N/A – Not Available

**Table 5.** WRF-Chem simulated BC mass mixing ratio in snow (μg kg^−1^) for the four months simulated in 2013 and the mean of those four months compared with the observed values reported in He et al. (2014). Himalayan sites are in normal text, sites in the Tibetan plateau or north of the plateau are in italics. The closest corresponding model month to the observation is in bold.

Location	Obs. Time	Lat. (°N)	Long. (°E)	Elev. (km)	This Work					Obs.	Ref.
					Jan.	Apr.	Jul.	Oct.	Mean		
Zuoquipu	monsoon 2006	29.21	96.92	5.5	73.3	3.7	**4.8**	1.6	4.2	7.9	2
	non-monsoon 2006	29.21	96.92	5.5	**73.3**	3.7	4.8	1.6	4.2	15.9	2
Qiangyong	summer 2001	28.83	90.25	5.4	4525	24.7	**2.9**	24.3	24.1	43.1	1
Noijin Kangsang	annual 2005	29.09	90.2	5.95	189	85.4	2.4	72.1	**42.5**	30.6	2
East Rongbuk	monsoon 2001	28.02	86.96	6.5	39.9	73.9	**4.9**	58.4	28.3	35	3
	non-monsoon 2001	28.02	86.96	6.5	**39.9**	73.9	4.9	58.4	28.3	21	3
	summer 2002	28.02	86.96	6.5	39.9	73.9	**4.9**	58.4	28.3	20.3	4
	Oct. 2004	28.02	86.96	6.5	39.9	73.9	4.9	**58.4**	28.3	18	4
	Sept. 2006	28.02	86.96	6.5	39.9	73.9	4.9	**58.4**	28.3	9	7
	May 2007	28.02	86.96	6.52	39.9	**73.9**	4.9	58.4	28.3	41.8	6
Kangwure	summer 2001	28.47	85.82	6	30.3	102	**5.4**	8163	32.0	21.8	1
Namunani	summer 2004	30.45	81.27	5.9	5.5	57	**1.2**	137	7.4	4.3	1
*Mt. Muztagh*	*summer 2001*	*38.28*	*75.02*	*6.35*	*31.9*	*19*	***93.1***	*51*	*34.0*	*32.9*	*1*
	*1999*	*38.28*	*75.02*	*6.35*	*31.9*	*19*	*93.1*	*51*	***34.0***	*42.1*	*1*
*Laohugou #12*	*Oct. 2005*	*39.43*	*96.56*	*5.05*	*37.8*	*190*	*27.5*	***360.5***	*38.9*	*65*	*4*
*Qiyi*	*Jul. 2005*	*39.23*	*97.06*	*4.85*	*218*	*615*	***14.6***	*3696*	*38.5*	*48.9*	*4*
*1 July Glacier*	*summer 2001*	*39.23*	*97.75*	*4.6*	*34.2*	*158*	***23.8***	*46285*	*34.4*	*106.2*	*1*
*Meikuang*	*summer 2001*	*35.67*	*94.18*	*5.2*	*11.5*	*18.4*	***7.8***	*22.9*	*12.0*	*32.9*	*1*
	*Nov. 2005*	*35.67*	*94.18*	*5.2*	*31.9*	*18.4*	*7.8*	***22.9***	*5.8*	*38.6*	*5*
*Tanggula*	*2003*	*33.11*	*92.09*	*5.8*	*13.7*	*4.9*	*2.8*	*18.7*	***5.8***	*12*	*2*
*Dongkemadi*	*summer 2001*	*33.1*	*92.08*	*5.6*	*23.5*	*4.6*	***3.9***	*28.6*	*8.2*	*15*	*1*
	*year 2005*	*33.1*	*92.08*	*5.6*	*23.5*	*4.6*	*3.9*	*28.6*	***8.2***	*11.8*	*7*
*La’nong*	*Jun. 2005*	*30.42*	*90.57*	*5.85*	*120*	*9.9*	***3.1***	*70.7*	*11.2*	*22.9*	*4*
*Zhadang*	*Jul. 2006*	*30.47*	*90.5*	*5.8*	*120*	*9.9*	***3.1***	*70.7*	*11.2*	*19.3*	*4*
*Haxilegen River*	*Oct. 2006*	*43.73*	*84.46*	*3.76*	*227*	*19.2*	*23.7*	***978***	*35.7*	*46.9*	*4*
*Urumqi Riverhead*	*Nov. 2006*	*43.1*	*86.82*	*4.05*	*116*	*12.9*	*15.7*	***353***	*19.3*	*141*	*5*
*Miao’ergou #3*	*Aug. 2005*	*43.06*	*94.32*	*4.51*	*222*	*352*	***31.7***	*9161*	*56.3*	*111*	*4*

References: [1] Xu et al. (2006), [2] Xu et al. (2009), [3] Ming et al. (2008), [4] Ming et al. (2009a), [5] Ming et al. (2009b), [6] Ming et al. (2012), [7] Ming et al. (2013).

**Table 6.** WRF-Chem simulated BC surface mass concentration (mean, μg m^−3^) averaged over the four months simulated for the moderate future ENSO case with two different emission scenarios - no further control (NFC) and mitigation (MIT). Also shown in italics is the percentage difference at each site for the MIT case versus NFC, calculated as (MIT-NFC)/NFC×100%. Results for 2013 are shown in Table 4.

Site Name	January			April			July			October		
	NFC	MIT	*Diff (%)*	NFC	MIT	*Diff (%)*	NFC	MIT	*Diff (%)*	NFC	MIT	*Diff (%)*
Dehli	18.3	5.9	*−68%*	10.4	3.7	*−64%*	5.8	1.5	*−74%*	14	4.2	*−70%*
Kanpur	5.7	2.1	*−63%*	3.3	1.1	*−67%*	1.6	0.35	*−78%*	4.2	0.81	*−81%*
Kharagpur	9.1	3.3	*−64%*	3.7	1.2	*−68%*	2.2	0.78	*−65%*	4	1	*−75%*
Dibrugarh	1.9	0.49	*−74%*	0.75	0.21	*−72%*	0.31	0.091	*−71%*	0.84	0.094	*−89%*
Trivandrum	2	0.6	*−70%*	1.4	0.28	*−80%*	0.52	0.14	*−73%*	1.6	0.51	*−68%*
Minicoy	0.11	0.1	*−9%*	0.026	0.026	*0%*	0.017	0.008	*−53%*	0.073	0.018	*−75%*
Port-Blair	0.43	0.15	*−65%*	0.019	0.013	*−32%*	0.002	0.001	*−50%*	0.004	0.002	*−50%*
**Nainital**	**1.6**	**0.7**	***−56%***	**1.4**	**0.66**	***−53%***	**0.69**	**0.16**	***−77%***	**1.5**	**0.35**	***−77%***
**Nagarkot**	**1.8**	**0.92**	***−49%***	**1.8**	**0.86**	***−52%***	**0.34**	**0.08**	***−76%***	**1.1**	**0.19**	***−83%***
**Lhasa**	**0.16**	**0.05**	***−69%***	**0.15**	**0.038**	***−75%***	**0.033**	**0.011**	***−67%***	**0.11**	**0.03**	***−73%***
**Langtang**	**0.44**	**0.27**	***−39%***	**0.53**	**0.22**	***−58%***	**0.095**	**0.021**	***−78%***	**0.3**	**0.054**	***−82%***
**NCO-P**	**0.11**	**0.078**	***−29%***	**0.14**	**0.033**	***−76%***	**0.032**	**0.007**	***−78%***	**0.069**	**0.012**	***−83%***

**Table 7.** WRF-Chem simulated BC surface mass concentration (mean, μg m^−3^) averaged over the four months simulated using the no further control (NFC) emission scenario to simulate the moderate ENSO (Mod), La Niña (La) and El Niño (El) cases.

Site Name	January			April			July			October		
	Mod	La	El	Mod	La	El	Mod	La	El	Mod	La	El
Dehli	18.3	19.3	18.3	10.4	10.9	10.4	5.8	6.6	4.7	14.0	10.4	13.1
Kanpur	5.7	7.3	6.4	3.3	3.3	3.4	1.6	1.6	1.2	4.2	2.6	2.7
Kharagpur	9.1	9.5	8.2	3.7	4.4	3.7	2.2	1.3	1.5	4.0	2.8	2.7
Dibrugarh	1.9	2.0	1.5	0.75	0.94	0.89	0.31	0.44	0.27	0.84	1.2	0.81
Trivandrum	2.0	1.8	2.1	1.4	3.2	2.2	0.52	0.39	0.51	1.6	1.7	2.1
Minicoy	0.11	0.49	0.094	0.026	0.034	0.022	0.017	0.014	0.012	0.073	0.032	0.051
Port-Blair	0.43	0.093	0.12	0.019	0.016	0.019	0.002	0.003	0.002	0.004	0.008	0.001
**Nainital**	**1.6**	**2.1**	**2.0**	**1.4**	**1.2**	**1.1**	**0.69**	**0.87**	**0.68**	**1.5**	**1.1**	**1.1**
**Nagarkot**	**1.8**	**3.1**	**1.9**	**1.8**	**1.8**	**1.3**	**0.34**	**0.24**	**0.49**	**1.1**	**0.45**	**0.50**
**Lhasa**	**0.16**	**0.21**	**0.22**	**0.15**	**0.14**	**0.13**	**0.033**	**0.028**	**0.036**	**0.11**	**0.096**	**0.087**
**Langtang**	**0.44**	**0.72**	**0.53**	**0.53**	**0.59**	**0.40**	**0.095**	**0.084**	**0.17**	**0.30**	**0.11**	**0.14**
**NCO-P**	**0.11**	**0.20**	**0.16**	**0.14**	**0.23**	**0.13**	**0.032**	**0.029**	**0.057**	**0.069**	**0.025**	**0.036**

**Table 8.** Total, in-domain anthropogenic (ANT), and boundary (BDY) BC deposition (Mg d^−1^) to the HKHK region (defined as areas with surface pressure less than 700 hPa) using the no further control (NFC) emission scenario to simulate the moderate ENSO, La Niña, and El Niño cases.

	Phase	January	April	July	October	Average
Total	Moderate	158.1	141.7	83.9	137.0	130.2
	La Niña	179.2	299.1	95.7	117.4	172.9
	El Niño	204.9	276.8	57.9	134.9	168.6
ANT	Moderate	86.0	63.6	46.4	66.4	65.6
	La Niña	110.7	168.3	55.2	58.2	98.1
	El Niño	135.9	179.7	22.7	61.9	100.0
BDY	Moderate	68.0	72.3	37.0	70.3	61.9
	La Niña	66.1	102.5	40.3	55.4	66.1
	El Niño	66.6	72.3	34.8	72.4	61.5

## Key Points:

- Sources outside South Asia have a similar impact on black carbon deposition to the glaciers as South Asian anthropogenic sources.
- If emissions in South Asia (but not elsewhere) are mitigated, the contribution from South Asian anthropogenic sources is reduced to 11–34%.
- The changes due to phase of ENSO are generally small and don’t follow a consistent pattern.
