# Supplementary material for: Sources of Black Carbon Deposition to the Himalayan Glaciers in Current and Future Climates
Source: J Geophys Res Atmos. Author manuscript; Available in PMC 2020 Jun 29. (PMC7323718; doi:10.1029/2018jd029049)
Supplement: 1 [file NIHMS1508415-supplement-1.pdf]

Journal of Geophysical Research - Atmospheres

Supporting Information for

Sources of Black Carbon Deposition to the Himalayan Glaciers in Current and Future Climates

Matthew J. Alvarado[1], Ekbordin Winijkul[1,*], Rebecca Adams-Selin[1], Eric Hunt[1], Christopher Brodowski[1], Chantelle R. Lonsdale[1], Drew T. Shindell[2], Gregory Faluvegi[3,4], Gary Kleiman[5], and Thomas M. Mosier[5]

[1]Atmospheric and Environmental Research, Lexington, MA, USA
[2]Nicholas School of the Environment, Duke University, Durham, NC, USA
[3]Center for Climate Systems Research (CCSR), Columbia University, New York, NY, USA
[4]NASA Goddard Institute for Space Studies, New York, NY, USA
[5]World Bank Group, Washington, DC, USA
[*] Now at Department of Energy, Environment, and Climate Change, Asian Institute of Technology (AIT), Klongluang, Pathumthani, Thailand

Contents of this file

Figures S1 to S4

Introduction

This file contains maps of the fraction of the *in-domain anthropogenic* (not total) deposition of BC that is due to anthropogenic sources in the six different countries studied (Figures S1 to S4).

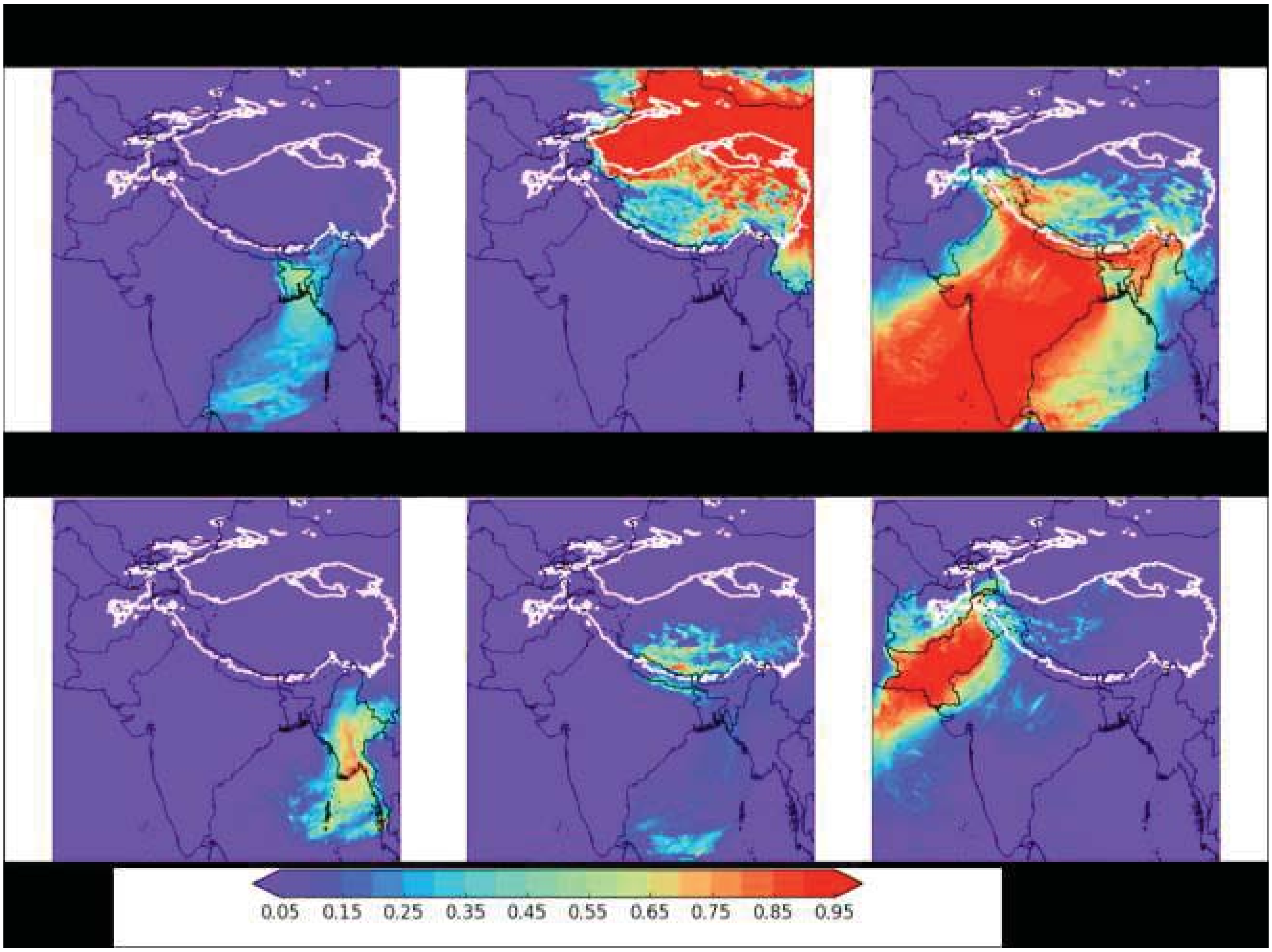

Figure S1. Fractions of the *in-domain anthropogenic* deposition of BC from (clockwise from upper center) Bangladesh, China, India, Myamnar, Nepal, and Pakistan in January 2013. Thick white contour shows the boundary of the 700 hPa surface pressure region.

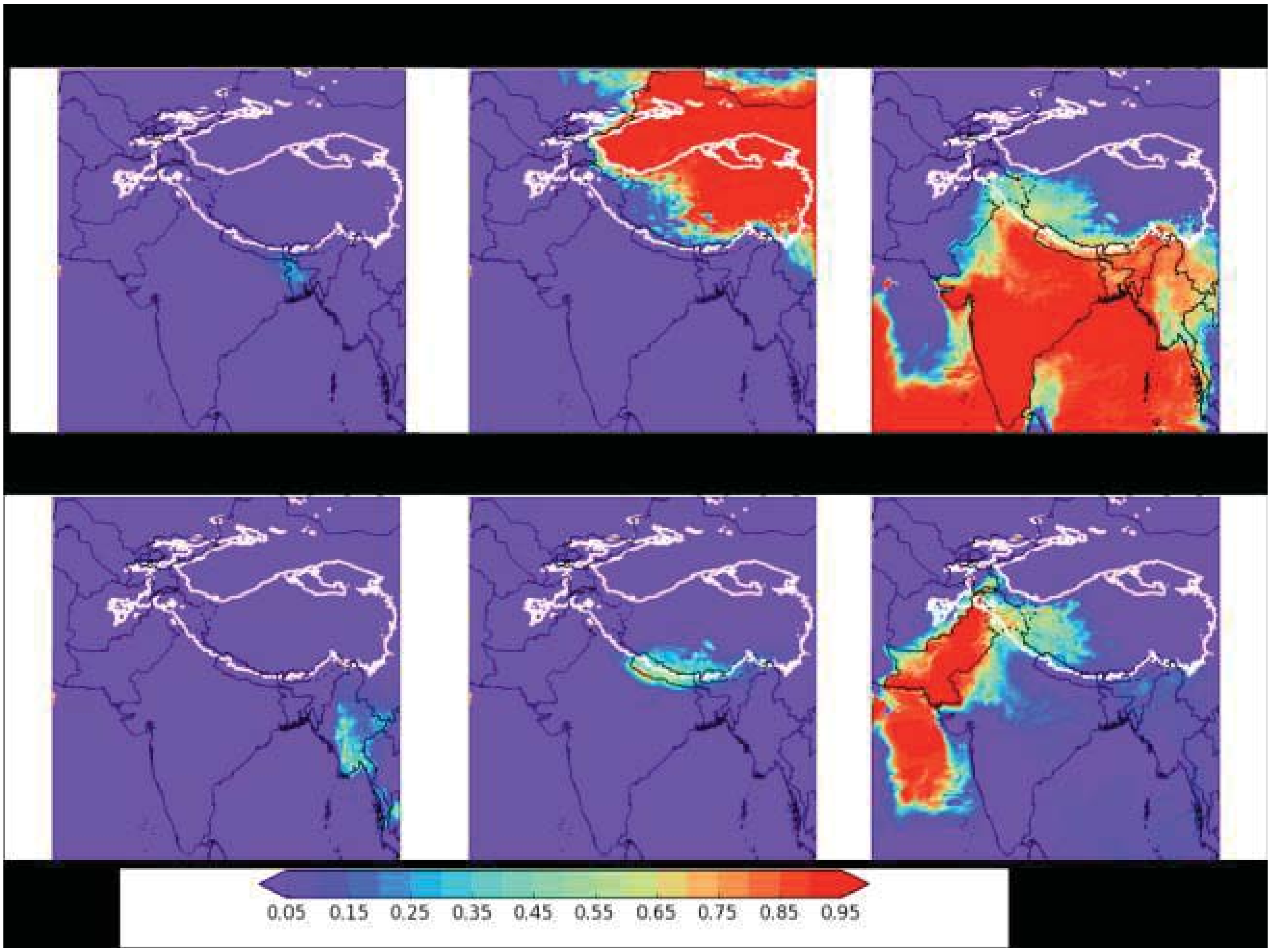

Figure S2. Fractions of the *in-domain anthropogenic* deposition of BC from (clockwise from upper center) Bangladesh, China, India, Myamnar, Nepal, and Pakistan in April 2013. Thick white contour shows the boundary of the 700 hPa surface pressure region.

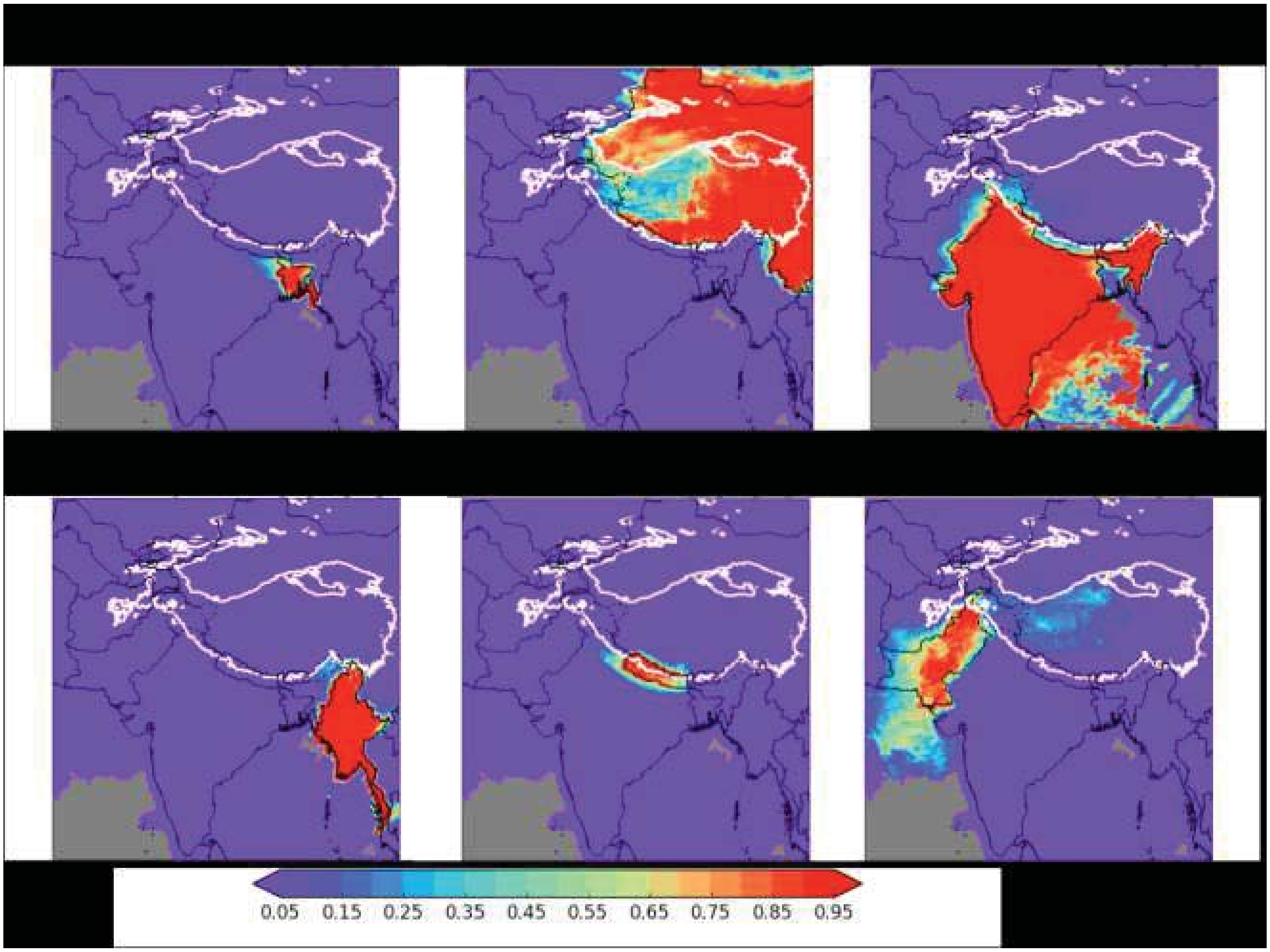

Figure S3. Fractions of the *in-domain anthropogenic* deposition of BC from (clockwise from upper center) Bangladesh, China, India, Myamnar, Nepal, and Pakistan in July 2013. Thick white contour shows the boundary of the 700 hPa surface pressure region.

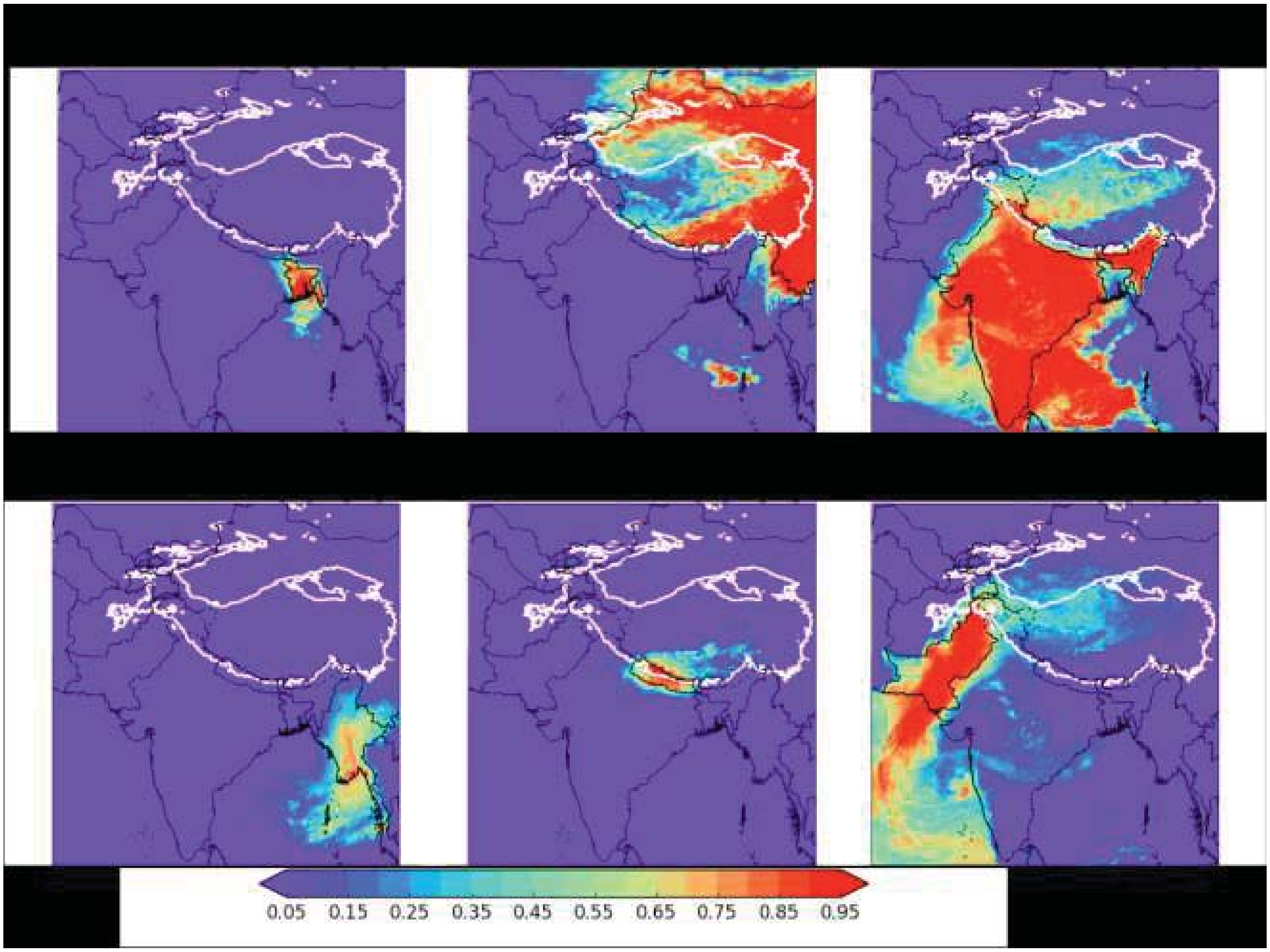

Figure S4. Fractions of the *in-domain anthropogenic* deposition of BC from (clockwise from upper center) Bangladesh, China, India, Myamnar, Nepal, and Pakistan in October 2013. Thick white contour shows the boundary of the 700 hPa surface pressure region.
